# Heat Wave and Bushfire Meteorology in New South Wales, Australia: Air Quality and Health Impacts

**DOI:** 10.3390/ijerph191610388

**Published:** 2022-08-20

**Authors:** Mohammad S. Islam, Tianxin Fang, Callum Oldfield, Puchanee Larpruenrudee, Hamidreza Mortazavy Beni, Md. M. Rahman, Shahid Husain, Yuantong Gu

**Affiliations:** 1 School of Mechanical and Mechatronic Engineering, University of Technology Sydney (UTS), 15 Broadway, Ultimo, NSW 2007, Australia; 2 Department of Biomedical Engineering, Arsanjan Branch, Islamic Azad University, Arsanjan 6134937333, Iran; 3 School of Computing, Engineering, and Mathematics, Western Sydney University, Penrith, NSW 2751, Australia; 4 Department of Mechanical Engineering, Zakir Husain College of Engineering & Technology, Aligarh Muslim University, Aligarh 202001, India; 5 School of Mechanical, Medical and Process Engineering, Faculty of Engineering, Queensland University of Technology, Brisbane, QLD 4000, Australia

**Keywords:** heat wave, bushfire, PM_10_, PM_2.5_, health impacts

## Abstract

The depletion of air quality is a major problem that is faced around the globe. In Australia, the pollutants emitted by bushfires play an important role in making the air polluted. These pollutants in the air result in many adverse impacts on the environment. This paper analysed the air pollution from the bushfires from November 2019 to July 2020 and identified how it affects the human respiratory system. The bush fires burnt over 13 million hectares, destroying over 2400 buildings. While these immediate effects were devastating, the long-term effects were just as devastating, with air pollution causing thousands of people to be admitted to hospitals and emergency departments because of respiratory complications. The pollutant that caused most of the health effects throughout Australia was Particulate Matter (PM) PM_2.5_ and PM_10_. Data collection and analysis were covered in this paper to illustrate where and when PM_2.5_ and PM_10,_ and other pollutants were at their most concerning levels. Susceptible areas were identified by analysing environmental factors such as temperature and wind speed. The study identified how these pollutants in the air vary from region to region in the same time interval. This study also focused on how these pollutant distributions vary according to the temperature, which helps to determine the relationship between the heatwave and air quality. A computational model for PM_2.5_ aerosol transport to the realistic airways was also developed to understand the bushfire exhaust aerosol transport and deposition in airways. This study would improve the knowledge of the heat wave and bushfire meteorology and corresponding respiratory health impacts.

## 1. Introduction

The latest bushfire in Australia was unprecedented in scale and intensity and has led to extensive habitat loss and catastrophic loss of human and animal life. Between September 2019 and February 2020, New South Wales (NSW) endured catastrophic and uncontrollable bushfires. The peak was between late December and late January [1]. The fires burnt a total of 13.3 million hectares, destroying over 2400 buildings. Areas such as the Hunter Region, Blue Mountains, Hawkesbury, Sydney, South Coast, and the Snowy Mountains were some of the areas that were affected by the fires. Over 1 billion animals were killed, endangering some species to extinction, such as the koala [2,3,4]. Some of these deaths were directly caused by the fires, while others were caused by hazardous air quality [2,3,4,5]. The bushfires were caused by a combination of meteorological and climatic conditions, which led to a decrease in rain and a climb in temperatures. According to the Australian Bureau of Meteorology, 2017 and 2018 experienced two consecutive dry years with a deficient level of rainfall. The year 2019 now becomes the warmest year on average and the lowest year in air humidity on record [6].

In terms of the air quality inspection, the bushfire has severely deteriorated air quality in New South Wales [7]. Bushfire release many pollutants, such as PM_2.5_, PM_10_, NO, NO_2_, CO, and Ozone [8,9,10]. These pollutants are exposed to urban air for a long time, and the urban population density is large. The particles are inhaled into the human respiratory tract, which causes many respiratory diseases. Apart from this, the high-intensity continuous and long-term burning of forest fires will produce heatwave effects [10,11,12,13]. With the increasing number of bushfire pollutants exposure, the burden of bushfire events on human health will also increase considerably [14]. The analysis of air quality and its impacts on health attracts the attention of the researchers after the bushfire between 2019 and 2020 in Australia. The Air Quality Index (AQI) in normal conditions is between 0 to 50 [15,16,17]. During the bush fire crisis, the AQI was measured five times higher than the “hazardous” level, with hazardous being defined as an AQI of over 300 [7]. The AQI index reported by the Weather Bureau can be used to predict when an area will reach severe levels of air quality. Firefighters and other emergency services will be able to advise and help the public and take more precautions, such as playing less outdoors and staying indoors to reduce the risk of respiratory problems [18,19,20,21,22,23,24]. The main pollutants that were generated from the bush fires and caused these respiratory problems were fine Particulate Matter 2.5 (PM_2.5_) and coarse Particulate Matter 10 (PM_10_) [24]. Moreover, the formula to calculate AQI, is inclusive these pollutants, as well as carbon monoxide, nitrogen dioxide, and ground-level Ozone. Each of these pollutants has its own AQI number. The overall AQI number is the highest of these calculations [18,19,20,21,22,23,24].

Furthermore, PM_2.5_ and PM_10_ are a combination of solid and liquid particles suspended in the air. The number (2.5 or 10) refers to the diameter of the particle in micrometres [25,26,27]. Under normal circumstances, particles with a particle size of smaller than 10 microns can be inhaled by the human respiratory tract along with the airflow and deposited in the lungs. Particles with a particle size of less than 2.5 microns can even be deposited in the lower respiratory tract [28]. These pollutants are a result of burning fuels and chemical reactions. Since PM_2.5_ particles are so small, they tend to stay in the air longer than PM_10_ particles [26]. This, therefore, increases the risk of humans and animals inhaling them into the body [27]. Due to their small size, the particles can bypass the nose and throat, and then penetrate deep into the lungs and possibly into the circulatory system causing problems such as heart disease, asthma and/or chronic bronchitis. Given the individual’s current conditions, the intake of large amounts of PM_2.5_ particles can ultimately lead to death [29,30,31,32]. Depending on the source, particulate matter (PM) particles can vary in density and can travel for thousands of kilometres. The distance travelled factors include temperature, wind, dryness, and terrain [33,34].

Therefore, this paper will analyse the temperature and wind speed data, and study the change rule of heat wave and air quality through the change of pollutants in different regions within the same time interval. In addition, based on the transport and deposition of bushfire exhaust aerosols in human airways, this paper will infer the impact of meteorological changes in heat waves on respiratory diseases. This paper will collect main variables including average temperature [°C], wind speed [m/s], Nitric Oxide—NO [pphm], Nitrogen Dioxide—NO_2_ [pphm], Carbon Monoxide [ppm], Ozone—O_3_ [pphm], PM_10_ [µg/m^3^], PM_2.5_ [µg/m^3^] monthly and daily at various locations of Sydney Central East, North West, South West, and Upper Hunter from November to June during 2018–2019 and 2019–2020. This paper will discuss the trends of these variables separately for the horizontal-timeline and vertical-different air pollutants directions. Moreover, this paper will also compare the number and crude rate of admitted patient hospitalisations in 2019–2020 with the previous five years to provide more details. The analysis of the bush fires and their causes from September 2019 to February 2020 will allow us to get a better understanding of PM particles, including how far they can travel as well as how long they can stay in the air and to what extent they can cause harm to humans and animals.

## 2. Methodology

The first step of the methodology is to analyse the relevant literature critically. These literature reviews covered a broad range of studies, some of which looked into the environmental factors that heighten the levels of PM_10_ and PM_2.5_, while others looked into what happens when the particulate matter enters the respiratory system. For an effective literature review, the University of Technology Sydney Library database and Google Scholar are used as search engines. Firstly, the heat wave and bush fire-related literature are searched from the database. Secondly, the literature on PM_10_ and PM_2.5_ and associated health impacts are collected.

**Figure 1:**
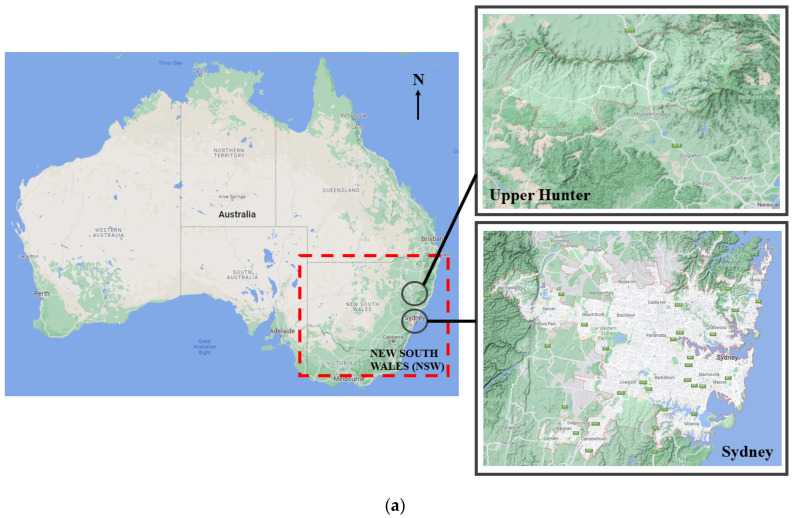

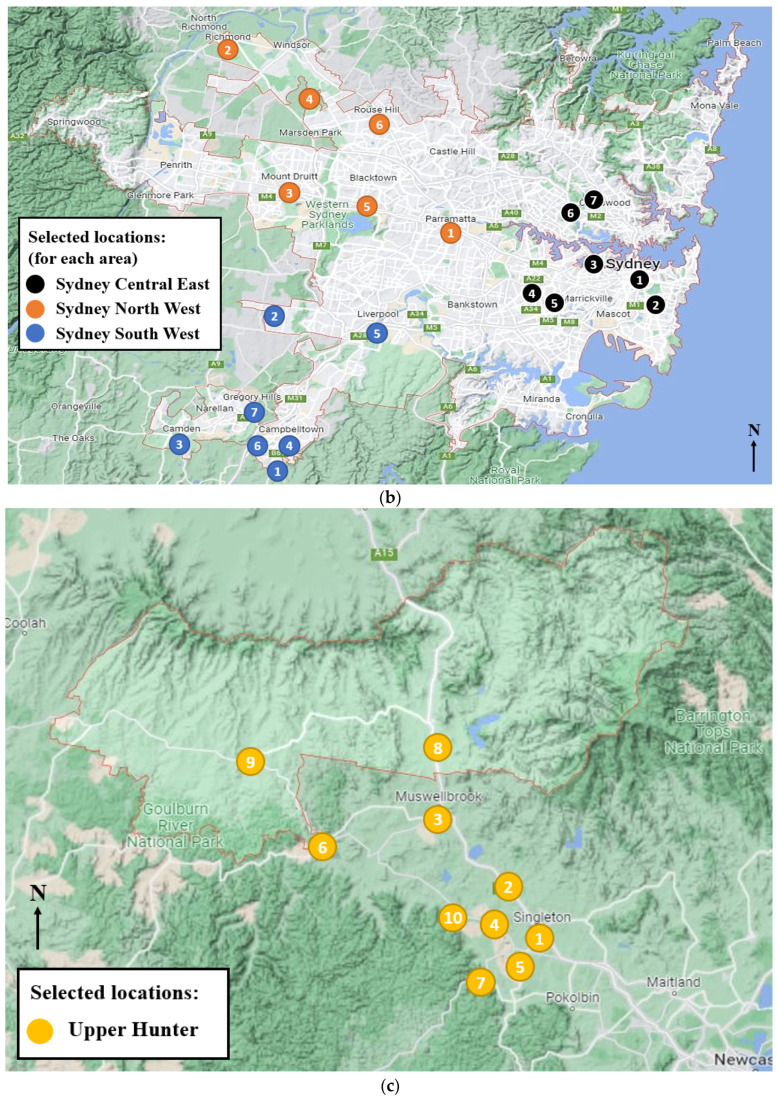
The location map of the sight of the air-quality analysis. (**a**) selected areas including Upper Hunter and Sydney, (**b**) selected sight of air-quality analysis in Sydney, and (**c**) selected sight of air-quality analysis in Upper Hunter. (All numbers are referred to the selected locations which can be seen in Table 1).

Apart from the literature reviews, the project’s first task is to collect air-quality data for the selected time periods. Implementing this study needed a comparative timeframe, recording data over the time of the Australian bushfires (2019–2020) and comparing it with a year that did not have catastrophic fires (2018–2019). Due to insufficient data for selected substances and pollutants that were unavailable in several previous years before catastrophic fires, the present study only considers two periods between 2018–2019 and 2019–2020. By developing graphical models, trends will be identified and determine what factors may have contributed to the fires in 2019–2020. The regions of the study would include areas in Sydney Central East, Sydney North West, Sydney South West, and Upper Hunter. Figure 1 and Table 1 illustrate the areas with weather stations providing data.

The environmental variables that will be collected from these areas include Temperature [°C], Wind speed [m/s], Nitric Oxide—NO [pphm], Nitrogen Dioxide—NO_2_ [pphm], Carbon Monoxide—CO [ppm], Ozone—O_3_ [pphm], PM_10_ [µg/m^3^], and PM_2.5_ [µg/m^3^]. In the presence of lightning or a spark, nitrogen combines with oxygen to form several different oxides. NO and NO_2_ are the most abundant, which are two kinds of gases referred to as nitrogen oxides (NOx). To provide a comprehensive evaluation, NO and NO2 are considered and present separately in this study.

The data obtained are from hourly data from each suburb. From this, the data are sorted in Excel, and organised in terms of daily and monthly data. A comprehensive analysis is performed for different environmental variables. The average, maximum, and minimum data for the hourly and monthly basis will be calculated. The health data during the bushfire period will be collected, and it will be compared with the previous five years’ average health data. All air quality and meteorological data have been collected from NSW government’s department of planning and environment (DPIE) (https://www.dpie.nsw.gov.au/air-quality/air-quality-data-services, accessed on 19 November 2020). The health data are collected from the Australian Institute of Health and Welfare database (https://www.aihw.gov.au/reports/environment-and-health/data-update-health-impacts-2019-20-bushfires/data, accessed on 17 May 2022). The earth satellite images are collected from the NASA Goddard Space Flight Center website (https://giovanni.gsfc.nasa.gov/giovanni/, accessed on 5 March 2022). Goddard Space Flight center is one of the leading space research lab of NASA. The space centre is located in Maryland, United States. The study only analysed the surface temperature and CO emission images from the centre.

### 2.1. Bushfire Periods 2019–2020

The Australian Black Summer bushfires first started from a lightning strike in Wollumbi National Park. This was the Gospers mountain fire. It raged for a total of 79 days, burning to the edge of Sydney and threatening suburban areas. It was only 2.5 h after ignition when the fire had spread 65 hectares. As firefighters were trying their best to put out the fire, wind speeds reached about 67 km/h, ultimately being too windy for helicopters to spray the water into the flames. Therefore, fixed-wing aircraft were brought it to water bomb the fire. On 31 October, the rain had extinguished most of the Gospers Mountain fire. However, on 7 November, the fires started again and doubled in size in one day. On 12 November, the fire had jumped fire breakers at Putty Road, resulting in the premiere declaring a state of emergency. On this day, the flames had traversed 12 km over 2.5 h, with over 56,000 hectares being burnt in total. On 24 November, storms had formed outside the fire control perimeter and created three more fires, including the Three Mile Creek fire, Little L complex fire, and Thompsons Creek fire.

On 3 December, the weather conditions were a lot calmer, and firefighters had strategized to extinguish the fire once and for all. However, two people were declared missing during the bushfires. Thus, a search and rescue mission commenced. The people were found, but the firefighters had lost 17 h of work in which the fire had already spread and was again out of control. On 6 December, computer simulations suggested that the fires could merge into one, forming a mega-fire. Unfortunately, these haunting predictions became a reality. Gospers mountain fire merged with the Little L complex fire and the Paddock run fire. Later that day, the Thompsons creek fire had also merged. At this point, the mega fire had been formed; the peak was on 21 December. On 8 January, the mega fire had finally been contained and under control. However, it took over a month of hard work from firefighters and flooding to extinguish the fire completely.

### 2.2. Data Analysis

#### Expected Results

It is evident from the literature [8,9,10] that bushfire produces a lot of toxic gas and aerosols, significantly affecting air quality. Therefore, measurements of the different meteorological variables and air quality parameters are expected to be higher than in previous years.

Before obtaining the environmental data from the government database, it is first necessary to select the important air quality and meteorological variables related to the heat wave and bush fire. This establishes the significance of the study based on the proposed methodology, the timeline of events and the analysis of the literature reviews. By identifying the dates of when the fires had started, and days of severe weather, we could predict environmental factors such as temperature, PM_10_, and PM_2.5_ levels. From knowing when the bushfires occurred and researching the by-products of bushfires, a better understanding of which pollutants would be more prominent within the bushfire period. Bush fires could produce toxic air pollution such as PM_2.5_, PM_10_, carbon monoxide, carbon dioxide, and nitrogen oxides. Therefore, it is expected that the levels of these pollutants in 2019–2020 will be far greater than in the 2018–2019 period, especially in November, December, and January. According to Figure 1, it can be seen that the terrain in Upper Hunter is different from other areas in Sydney (populated areas). Furthermore, due to the distinctive maritime influences from the Pacific Ocean (the northerly latitude and close oceanic influences), Upper Hunter is one of Australia’s hottest and wettest regions [35]. Therefore, it is expected that the trends of all selected parameters from these regions will be significantly different from other areas in Sydney.

Another expected trend would be based on the end of the bushfires. This was around the beginning of February. Because of the torrential rain and flooding, the expectation of the PM_2.5_ and PM_10_ levels was to drop. Another reason for these pollutants to drop in levels is also because of the COVID-19 pandemic. It was also around February–March that the pandemic had reached Australia. As a result of this, lockdown restrictions were put in place by the government. Because of this, there were fewer vehicles on the road and not as many industrial companies continuing operations. These are both sources of many pollutants, including particle pollution, ground-level Ozone, carbon dioxide, sulphur oxides, and nitrogen oxides. Therefore, it is expected that all these pollutants’ levels would reduce significantly in February and March.

### 2.3. Computational Model

According to Hosker [36] and Pesic et al. [37], the local wind fields and air pollutants transport and dispersion could be influenced by the buildings, including isolated buildings, building clusters, and urban street canyons. Therefore, several methods have been used to analyse and estimate air pollutants in several areas. These include the urban areas, power plants, as well as development of industry [38,39,40,41]. However, to understand the basic concept of how the inhaled pollutant affects the human respiratory system, the last step of the study is to analyse the transport and deposition behaviour of PM_2.5_ in healthy and diseased airways. The lung model was developed based on the lung dimension from Weibel’s model [42]

ANSYS Fluent 2021 solver is used for the computational purpose. Steady mass and momentum equations are solved for the airflow and particle transport. The Ansys meshing module is used for the computational grid. The PM_2.5_ transport behaviour is analysed for the heavy activity physical condition, and two different lung airway model is used for the simulation. The velocity inlet and pressure outlet boundary conditions are used for the calculations.

### 2.4. Obtaining the Results

The raw data was collected from the database of the department of planning and environment division, NSW government (https://www.dpie.nsw.gov.au/air-quality/air-quality-data-services, accessed on 19 November 2020). The data collection procedure followed three steps. Firstly, the data category and parameters were selected from the database. Hourly, daily, and monthly sight average data for the pollutants and meteorological variables are selected. Secondly, the data collection sites and stations are selected for different parts of the Sydney and Hunter region. Thirdly, the data tables are downloaded for the given range of periods. From the 10 environmental factors, the graphs illustrate the daily and monthly data in terms of the average value and the maximum value.

The raw data is then analysed by considering the period and range of each parameter. Then, the set of this data is presented as a chart using Microsoft Excel. A dynamic model is developed in this study. The data was then organised on a daily and monthly basis. For the monthly average data, the daily data for the whole month is collected at first, and the monthly average is calculated for all variables. For the daily average data, the information for 24 h is collected every day, and the average is calculated in Microsoft Excel. As observed, this process was completed for the time periods November 2018–July 2019 and November 2019–July 2020. All regions for each graph were plotted on one graph to compare the trends easily.

## 3. Results

The study analysed the bush fire exhaust pollutants and meteorological variables during the catastrophic bushfire session and COVID-19 lockdown period in 2019–2020 for different parts of NSW and compared with the previous year’s data. A wide range of pollutants and metrological variables are considered for the overall analysis.

### 3.1. Selected Parameters and Pollutants

#### 3.1.1. Average Temperature

**Figure 2:**
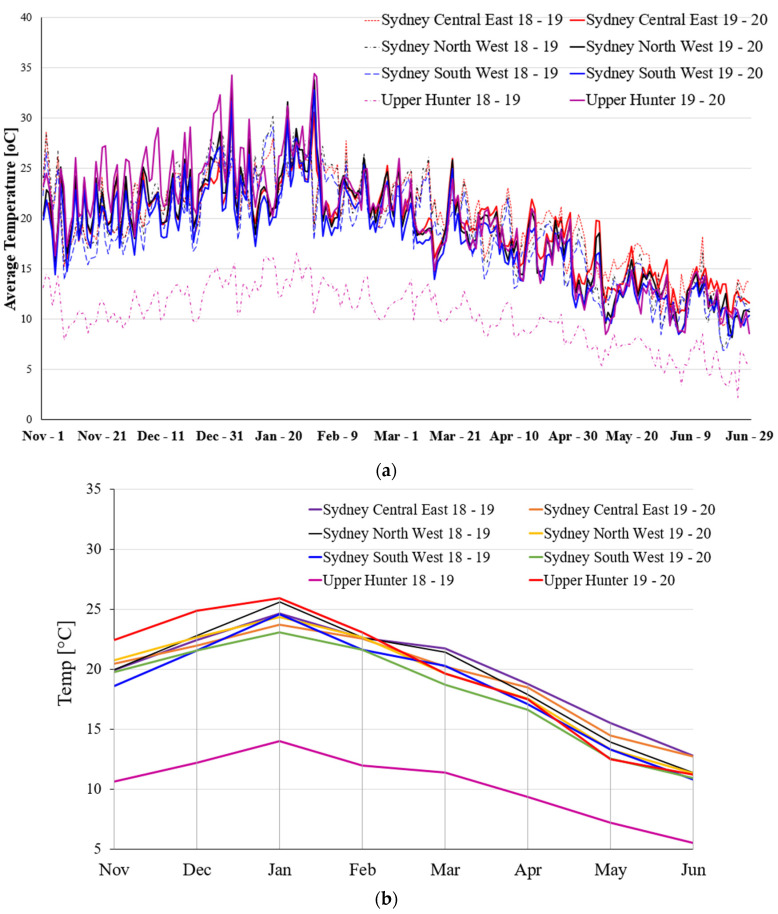
The average temperature (°C) in Sydney Central East, North West, South West, and Upper Hunter region for a period of eight months during 2018–2019 and 2019–2020, (**a**) daily average temperature, and (**b**) monthly average temperature.

Figure 2 shows the average daily and monthly temperatures in various selected regions of NSW. Figure 2a shows the daily average temperature for 8 months in the selected bushfire-affected regions of NSW. The overall temperature curve shows the highest temperature in the Upper Hunter region in 2019–2020 and the lowest average temperature in the same region in 2018–2019. The average daily temperature shows an increasing trend from the first week of November to the first week of February and it reached its peak during the first week of February in 2019–2020. The average daily temperature during this period is higher than in other selected regions. The overall average daily temperature shows a decreasing trend from the second week of February.

**Figure 3:**
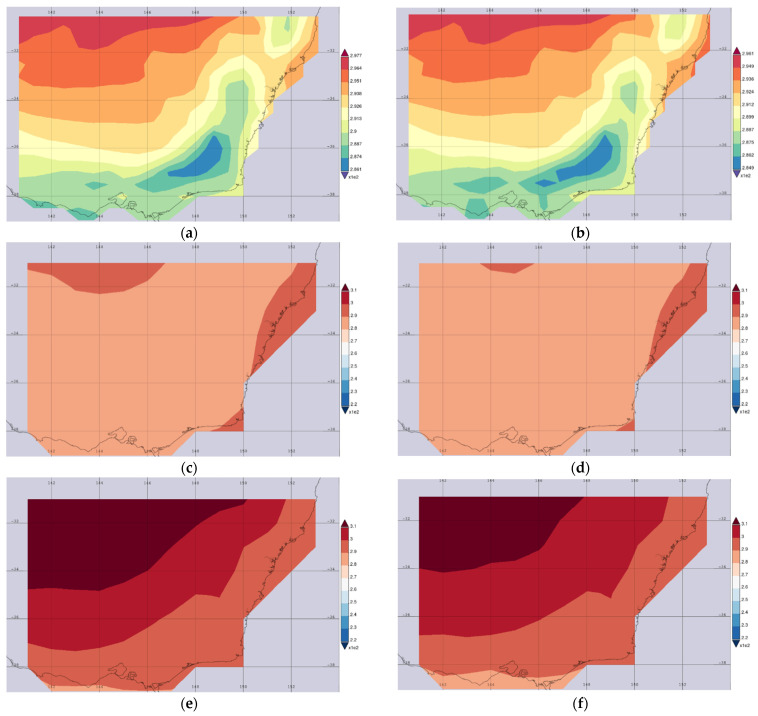
Time average map of surface temperature at NSW for day and night time for a period of eight months during 2018–2019 and 2019–2020, (**a**) overall monthly surface temperature 2018–2019, (**b**) overall monthly surface temperature 2019–2020, (**c**) nighttime descending 2018–2019, (**d**) nighttime descending 2019–2020, (**e**) daytime ascending 2018–2019, and (**f**) daytime ascending 2019–2020 (accessed on 23 April 2022).

Figure 3 shows the average map of surface air temperatures for the selected region (140.1153 E, 39.0527 S, 154.002 E, 30.0879 S). The earth satellite images are collected from the NASA Goddard Space Flight Center website (https://giovanni.gsfc.nasa.gov/giovanni/) during November to June 2018–2019 and 2019–2020. Figure 3a shows the average monthly surface air temperature over 2018-November to 2019 June and Figure 3b shows the average surface temperature over 2019-November to 2020 June. The satellite sensor MERRA-2 Model M2TMNXFLX v5.12.4 is used to capture the surface air temperature (0.5 × 0.625 deg.). The satellite sensor AIRS (Atmospheric Infrared Sounder) AIRS3STM v7.0 is used to collect the surface images for the surface air temperature of the selected region. Figure 3c shows the time-averaged nighttime descending temperature from November 2018 to June 2019. Figure 3d shows the nighttime descending temperature during November 2019 to June 2020. The overall average temperature at nighttime during a wide range of periods is found similar to the satellite images. The time-averaged daytime ascending temperature map is also captured through the satellite sensor AIRS AIRS3STD v7.0. Figure 3e, f show the daytime averaged temperature during 2018–2019 and 2019–2020, respectively. This figure shows that the overall monthly surface temperature from these periods in the Upper Hunter region (refer to Figure 1 for the locations) is higher than in other regions in Sydney.

**Figure 4:**
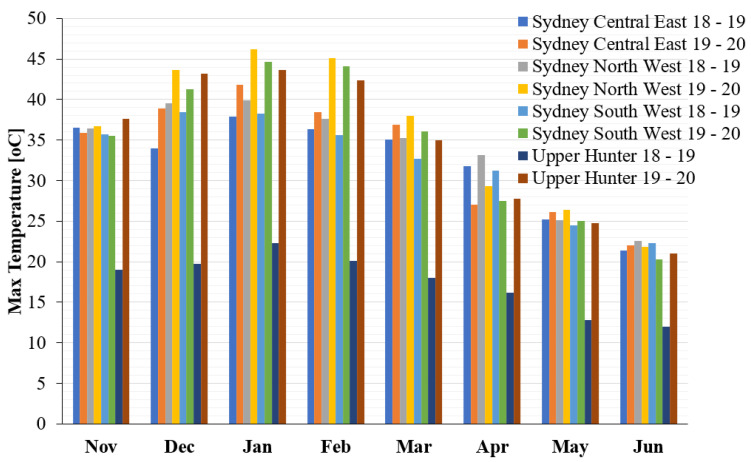
Maximum monthly temperature at different locations of NSW for a wide range of periods.

Figure 4 provides information about the maximum monthly temperature in different regions in NSW from November to June during 2018–2019 and 2019–2020. It is clear from the chart that the maximum monthly temperature among all locations in New South Wales is highest in January and lowest in June during 2018–2019 and 2019–2020, except in Upper Hunter where the maximum temperature in May and June was found to be similar.

#### 3.1.2. Average Wind Speed

**Figure 5:**
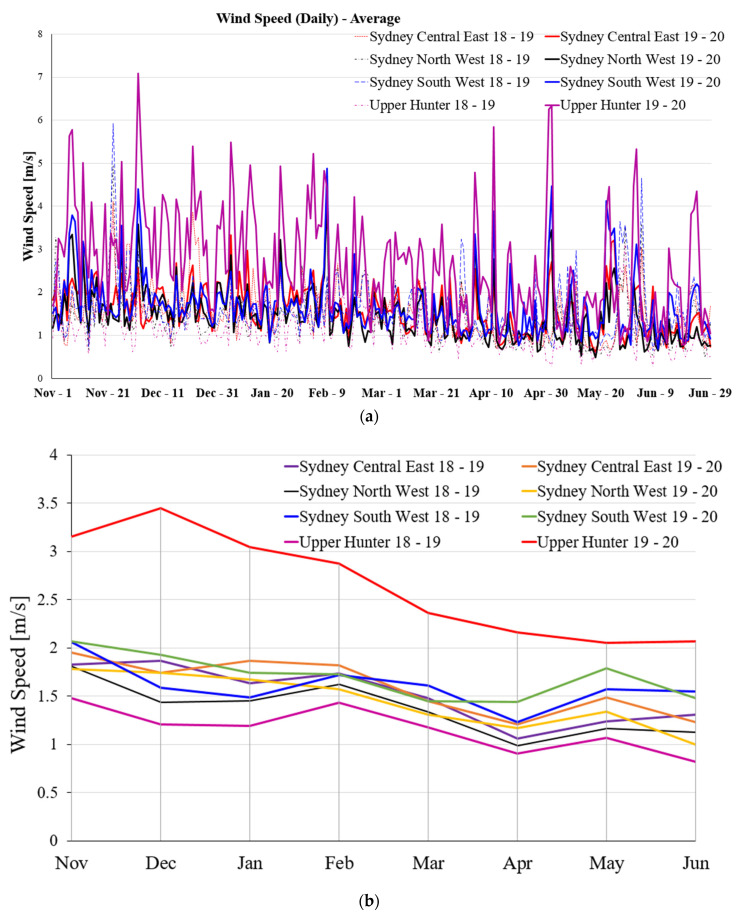
The average wind speed in Sydney Central East, North West, South West, and Upper Hunter region for a period of eight months during 2018–2019 and 2019–2020, (**a**) daily average wind speed, and (**b**) monthly average wind speed.

Figure 5 describes the average daily wind speed and average monthly wind speed at various selected locations of the NSW. Figure 5a illustrates the daily average wind speed for 8 months at the selected bushfire affected locations of the NSW. It is obvious that the average daily wind speed in Upper Hunter is the highest and during 2019–2020 compared to the other three locations in the same year. However, compared with the daily average wind speed in 2018–2019, there is always fluctuation among these four selected bushfire regions. Figure 5b illustrates the average monthly wind speed for the same regions during 2018–2019 and 2019–2020. From the Figure 5b, it shows that Upper Hunter has the highest average monthly wind speed compared with the other three selected bushfire regions in 2019–2020. For more information, the average solar and rain in these periods can be found in the supplementary file.

#### 3.1.3. Average Nitric Oxide (NO) Emission

**Figure 6:**
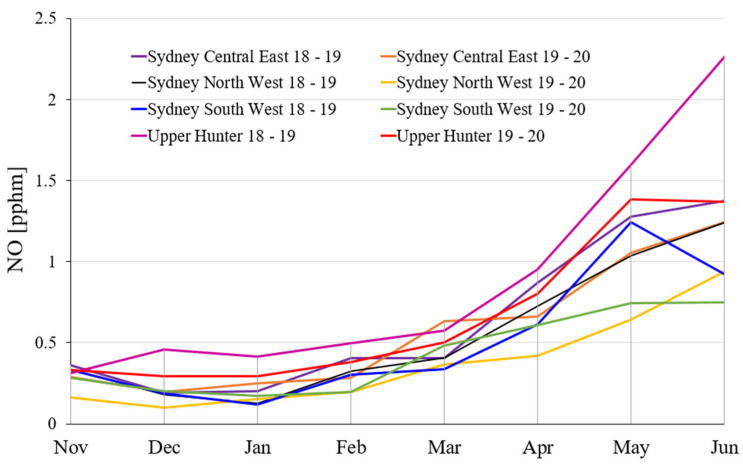
The monthly average NO emission in Sydney Central East, North West, South West, and Upper Hunter region for a period of eight months during 2018–2019 and 2019–2020.

Figure 6 shows the monthly average NO emission at the various selected regions in NSW for a period of 8 months during 2018–2019 and 2020. As demonstrated in Figure 6, from November to March during 2018–2019 and 2019–2020, the monthly average NO emission in the selected regions remains at a low level (not over 1 pphm). Different trends are seen over the period from March to June; the growths are marked during this time period.

The maximum NO emission on the various selected regions in NSW from November to next year June during 2018–2019 and 2019–2020 are calculated. The detail of the maximum NO emission can be found in the Table S1 in the Supplementary file.

#### 3.1.4. Average Nitrogen Dioxide (NO_2_) Emission

**Figure 7:**
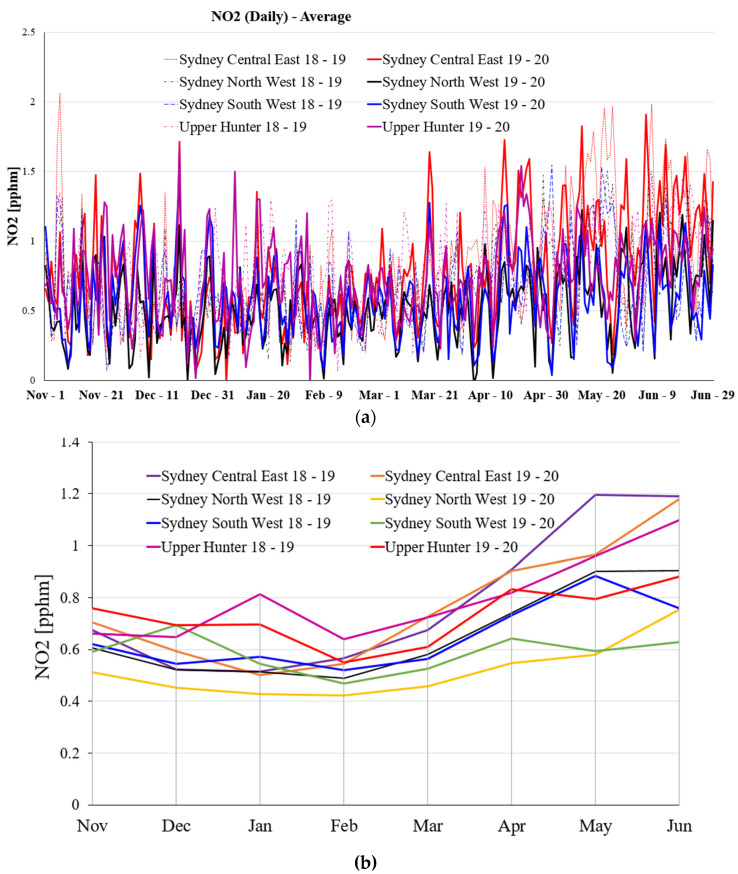
The average NO_2_ emission in Sydney Central East, North West, South West, and Upper Hunter region for a period of eight months during 2018–2019 and 2019–2020, (**a**) daily average NO_2_ emission, and (**b**) monthly average NO_2_ emission.

Figure 7 illustrates the daily average NO_2_ emission and monthly average NO_2_ emission at the various selected NSW regions for 8 months during 2018–2019, and 2020. Figure 7a presents the overall trend of daily average NO_2_ emission at the four selected locations in NSW within 8 months during 2018–2019 and 2019–2020. As is exhibited in Figure 7a, the daily average NO_2_ emission is lowest at the beginning of March among the 8 months during 2018–2019 and 2019–2020. It is clear from Figure 7a that the daily average NO_2_ emission in Sydney North West is lowest compared with the other three selected regions during 2019–2020. Figure 7b shows the monthly average NO_2_ emission trend in the selected regions in 8 months during 2018–2019 and 2019–2020. According to Figure 7b, the lowest monthly average NO_2_ emission always occurs in February among these 8 months during 2018–2019 and 2019–2020.

#### 3.1.5. Average Ozone Emission

**Figure 8:**
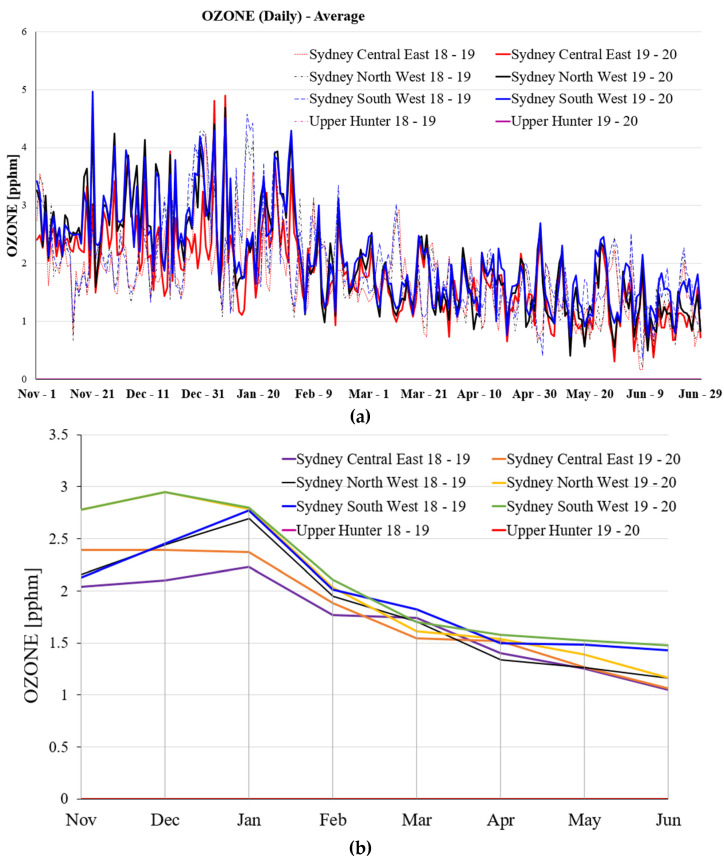
The average Ozone [pphm] in Sydney Central East, North West, South West, and Upper Hunter region for a period of eight months during 2018–2019 and 2019–2020, (**a**) daily average Ozone, and (**b**) monthly average Ozone.

Figure 8 illustrates the daily average Ozone and monthly average Ozone at the various selected locations in NSW for a period of 8 months during 2018–2019, and 2020. Figure 8a illustrates the daily average Ozone at selected locations, while the monthly average Ozone at selected locations is presented in Figure 8b. According to Figure 8a, the daily average Ozone in the four selected regions has declined dramatically since the middle of February during these selected years. The daily average Ozone from November to February is much higher than the daily average Ozone from March to June in these four selected regions these selected years.

The maximum ozone emission on the various selected regions in NSW from November to next year June during 2018–2019 and 2019–2020 are investigated. The detail of the maximum ozone emission can be found in Table S2 in the Supplementary file.

For more analysis, if considering the selected region in NSW from 2018–2019 to 2019–2020, it can be seen that the maximum ozone emission in Sydney Central East is significantly affected by bushfire in 2019–2020 compared to 2018–2019. However, if compared to the Month line, it can be clearly seen that the maximum ozone emission in these three selected regions is significantly affected by the bushfire from November to February. The monthly average Ozone in these four selected regions is highest from November to January, and the monthly average Ozone drops significantly from January to February, then there is a gradual decrease occurs from February to June during the period of 2018–2019 and 2019–2020. With regards to the comparison in same period of time during these selected years, the monthly average Ozone in Sydney South West is the highest among the other three selected regions. It can be seen that the average Ozone during 2019–2020 is higher than the average Ozone during 2018–2019 at all selected locations from December to January.

#### 3.1.6. Average Carbon Monoxide (CO) Emission

**Figure 9:**
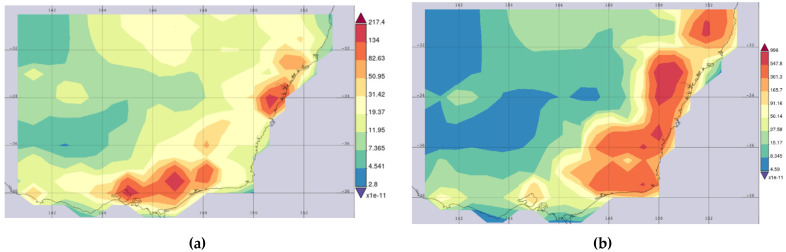
The average CO [kg/m^2^s] emission map on monthly basis over a wide range of periods, (**a**) November 2018 to June 2019, and (**b**) November 2019 to June 2020.

Figure 9 shows the average map of average CO emissions monthly for the selected regions (140.1153 E, 39.0527 S, 154.002 E, 30.0879 S). The earth satellite images are collected from the NASA Goddard Space Flight Center website (https://giovanni.gsfc.nasa.gov/giovanni/) during November to June 2018–2019 and 2019–2020. Figure 9a shows the map of average monthly CO emission over 2018-November to 2019 June and Figure 9b shows the average monthly CO emission over 2019-November to 2020 June. The satellite sensor MERRA-2 Model M2TMNXFLX v5.12.4 is used to capture the surface average CO emission (0.5 × 0.625 deg.). It should be noted that the range of average CO emission in 2018–2019 (Figure 9a) is 2.8–217.4 kg/m^2^s while the range of this emission in 2019–2020 (Figure 9b) is 4.59–996 kg/m^2^s. Focusing on the high range of the average CO emission (orange and red colours) for these two periods, the high range from 2018–2019 is 82.63–217 kg/m^2^s. However, the high range from 2019–2020 is 301–996 kg/m^2^s. Furthermore, it can be clearly seen that the amount of average CO emission has proliferated from November to June in 2019–2020 (more areas for red and orange colours) compared to the same period in 2018–2019.

Table 2 illustrates maximum CO emissions in the various selected NSW regions from November to June next year during 2018–2019 and 2019–2020. By comparison, it can be clearly seen that from November to February, the monthly maximum CO emission in the various selected regions in NSW during 2019–2020 is always higher than the monthly maximum CO emission during 2018–2019. In Sydney Central East for 2018–2019 and 2019–2020, the CO emission is similar in November and February, but there is a significant change between December and January, the maximum CO emission in Sydney Central East in 2019–2020 is, respectively, 1.8 times and 1.7 times higher than the data in December and January in 2018–2019. With regard to the comparison in same period of time during 2018–2019 and 2019–2020 from November to January, the maximum CO emission in Sydney North West in 2019–2020 is 3 times higher than the same date in 2018–2019. According to Table 2, the maximum CO emission in Sydney Central East and Sydney South West in May 2019–2020 is, respectively, 1.3 times and 1.4 times less than the same data in 2018–2019.

More information can be collected from Table 2, if considering the month line on selected region in NSW from 2018–2019 to 2019–2020, it can be seen that the CO emission is considerably affected by bushfires between November and February, as compared to 2018–2019, the CO emission in 2019–2020 shows an upward trend. However, when compared to the selected regions, Sydney North West is the most affected by the CO emission changes.

#### 3.1.7. Average PM_10_ Emission

**Figure 10:**
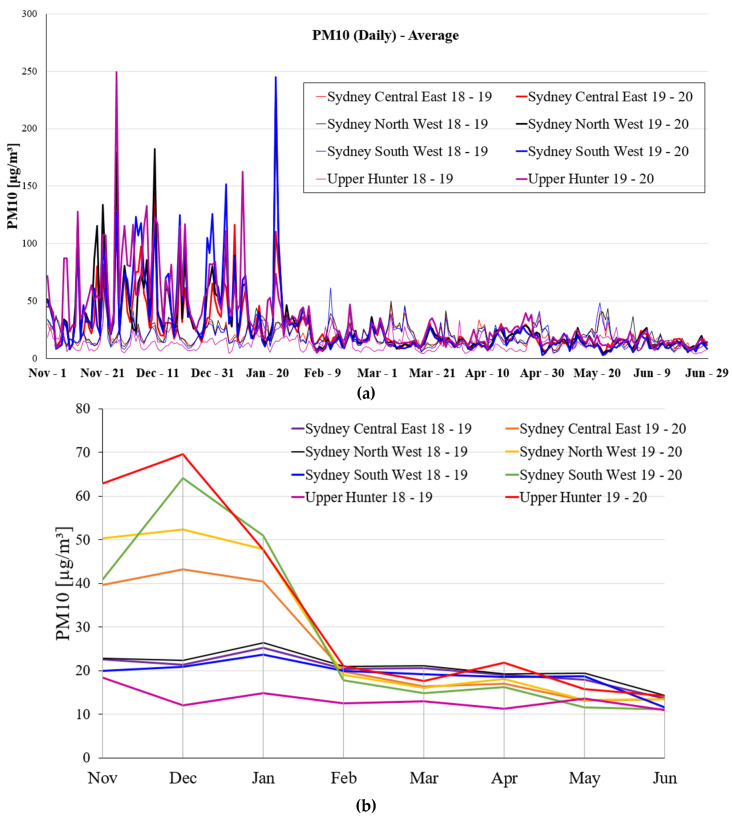
The average PM_10_ emission in Sydney Central East, North West, South West, and Upper Hunter region for a period of eight months during 2018–2019 and 2019–2020, (**a**) daily PM_10_ emission, and (**b**) monthly PM_10_ emission.

Figure 10 shows the daily average PM_10_ emission and monthly average PM_10_ emission at the various selected locations in NSW for 8 months during 2018–2019 and 2020. Figure 10a illustrates the overall trend of daily average PM_10_ emission at the four selected locations in NSW within 8 months during 2018–2019 and 2019–2020. According to Figure 10a, it can be seen that the daily average PM_10_ emission in Upper Hunter is highest in the third week of November and the daily average PM_10_ emission in Sydney South West is highest in third week of January during period of 2018–2019 and 2019–2020. The daily average PM_10_ emission in these four selected regions has a great fluctuation from November to January and there is a low fluctuation occurring from February to June during the period 2018–2019 and 2019–2020. Figure 10a shows that the daily average PM_10_ emission in these four selected regions has declined rapidly since the end of January. Figure 10b provides the information regarding the monthly average PM_10_ emission at the various selected locations in NSW for 8 months.

The monthly maximum emission on the four selected regions in NSW from November to next year June during 2018–2019 and 2019–2020 is calculated. The detail of the maximum PM_10_ emission can be found in Table S3 in the Supplementary file.

#### 3.1.8. Average PM_2.5_ Emission

**Figure 11:**
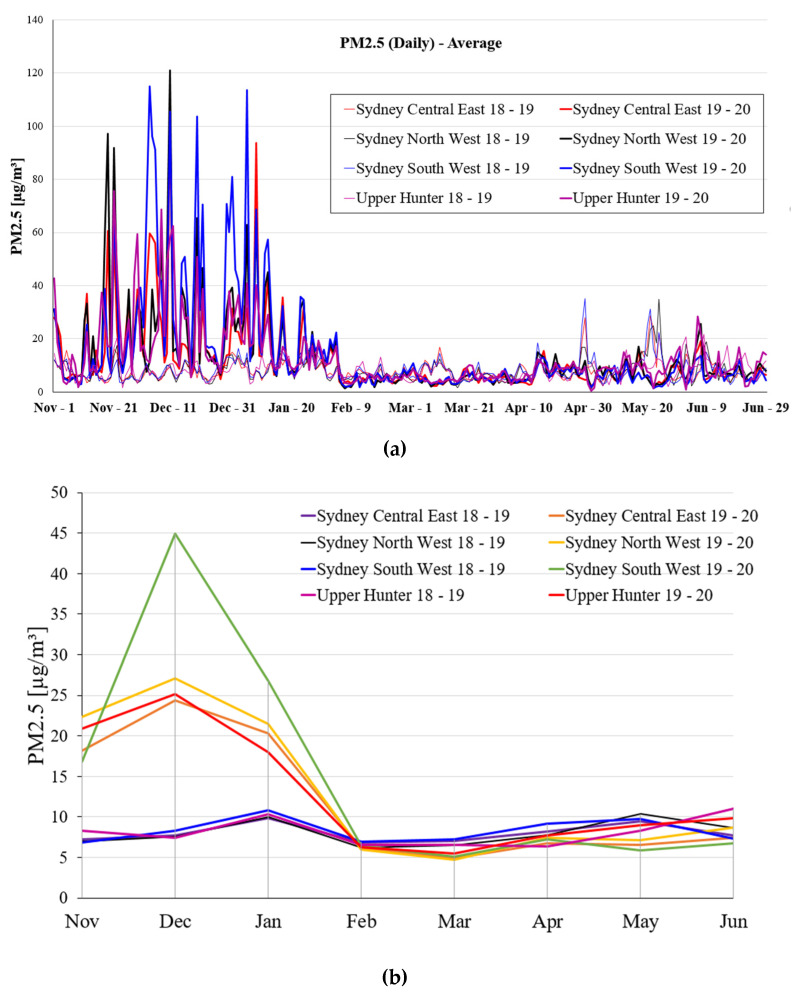
The average PM_2.5_ emission in Sydney Central East, North West, South West and Upper Hunter region for a period of eight months during 2018–2019 and 2019–2020, (**a**) daily PM_2.5_ emission, and (**b**) monthly PM_2.5_ emission.

Figure 11 shows the daily average PM_2.5_ emission and monthly average PM_2.5_ emission in the various selected regions in NSW for a period of 8 months during 2018–2019 and 2020. Figure 11a provides the information on the overall trend of daily average PM_2.5_ emission in the four selected regions in NSW within 8 months during 2018–2019 and 2019–2020. From Figure 11a, the daily average PM_2.5_ emission has a big fluctuation from November to the end of December and gentle fluctuations from January to June.

The information that monthly maximum PM_2.5_ emission on the various selected regions in NSW from November to next year June during 2018–2019 and 2019–2020 is calculated. The detail of the maximum PM_2.5_ emission can be found in Table S4 in the Supplementary file. It can be seen that the average PM_2.5_ emissions from all selected locations between November and January during 2019–2020 are significantly higher than the average PM_2.5_ emissions during 2018–2019. The trend of daily average PM_2.5_ emission and monthly average PM_2.5_ emission at the various selected regions in NSW from February to June during 2018–2019 and 2020 follows a similar trend. Meanwhile, the monthly maximum PM_2.5_ emission from March to May in 2019–2020 is always lower than the data during 2018–2019 among these four selected regions in NSW except Upper Hunter.

### 3.2. Health Impact

**Figure 12:**
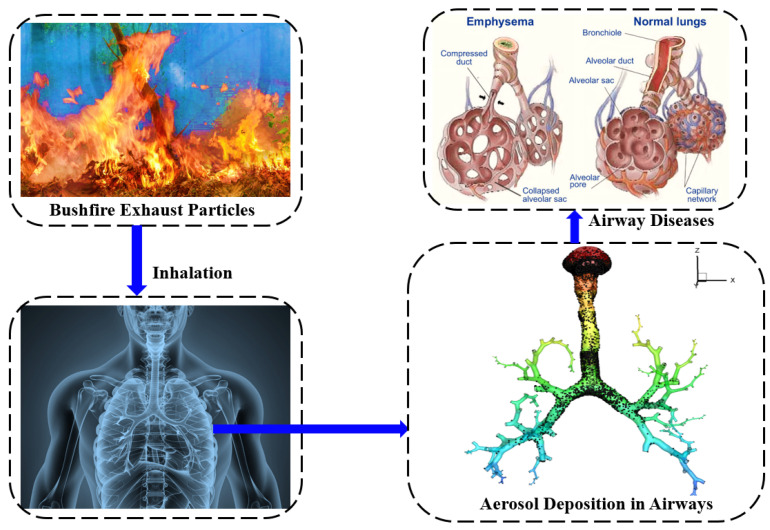
Respiratory health impacts of bushfire exhaust particles.

Figure 12 provides information on the evolution of bushfires on respiratory diseases. The healthy alveolar sac is a cavity surrounded by several adjacent alveoli that has the function of transporting nutrients [20]. Its essence is that the alveolar sac is composed of most unitary cells and is continuous with the alveolar tube, and each alveolar tube branches to form 2–3 alveolar sacs [21]. Bushfires produce a lot of smoke, such as carbon dioxide, carbon monoxide, hydrocarbons, carbides, nitrogen oxides, and particulate matter, which can stay in the air for a long time and are difficult to disperse. Particulate matter harms human health, such as inducing respiratory and chronic pulmonary heart diseases [17,19]. Bushfire exhaust particles and smoke are inhaled into human lungs. From the CT scan image, it can be seen that aerosols in the air will be deposited in the lungs. After this, this can cause a normal lung to become emphysema. Fine particulate matter is mainly a kind of pollutant, such as PM_2.5_ suspended particulate matter, which easily enters human respiratory tract in the air [17,19,21]. These particles will not be blocked by human’s respiratory, nasal, and oral cavities. These particles are easily inhaled into the trachea, bronchi, and alveoli, leading to many respiratory diseases, such as bronchial asthma, chronic bronchitis, and even chronic pneumoconiosis [19,21]. It may easily cause chronic bronchitis and emphysema, leading to chronic pulmonary heart disease [18,19,21].

Table 3 and Table 4 illustrates the number and crude rate of admitted patient hospitalisations, namely, Respiratory Conditions, Asthma, COPD (acute exacerbation), and Breathing Abnormalities during the 2019–2020 bushfire season and the previous 5 years’ average. The data is collected from the Australian Institute of health and Welfare database (https://www.aihw.gov.au/reports/environment-and-health/data-update-health-impacts-2019-20-bushfires/data).

#### 3.2.1. Computational Analysis

This study computationally analysed PM_2.5_ transport behaviour in lung. During inhalation, PM_2.5_ can penetrate deep into the lungs, where it may reach the blood capillaries unfiltered [43]. On the other hand, this phenomenon can potentially induce heart attacks, respiratory diseases, and early death [44]. The human airways with normal (healthy lung) and abnormal (Stenosis airways) are considered for the analysis.

##### Geometrical Development and Boundary Conditions

**Figure 13:**
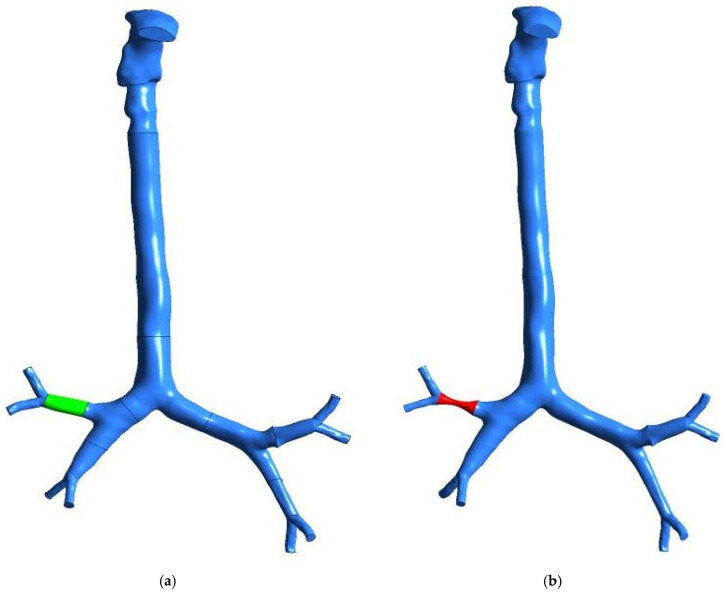
Reconstructed models of the mouth–throat and tracheobronchial airways: (**a**) healthy lung model, (**b**) stenosis lung model.

CT scans are used for the airway anatomical model in this investigation. The computational model consists of the mouth–throat and upper airways. Figure 13 depicts the reconstructed anatomical models with the same number of generations. The first model depicts a healthy lung with no abnormalities (Figure 13a). In contrast, the second model depicts pulmonary stenosis (which causes the lobe to shrink to 25% of its original size), represented in the right lobe (Figure 13b). With a smooth wall surface, the stenosis portion is constructed.

PM_2.5_ is injected from the mouth–throat surface of the model at the 60 L/min flow rate. The inlet velocity and outlet outflow conditions are used as boundary conditions [45]. In addition, the conditions of stationary walls and no-slip are applied to the airway walls. A ‘trap’ boundary condition is also used as a Discrete Phase Model (DPM) wall condition [46,47]. As a result of the trap conditions, particles should be deposited when the particle touches the lung wall.

##### Airflow Analysis

**Figure 14:**
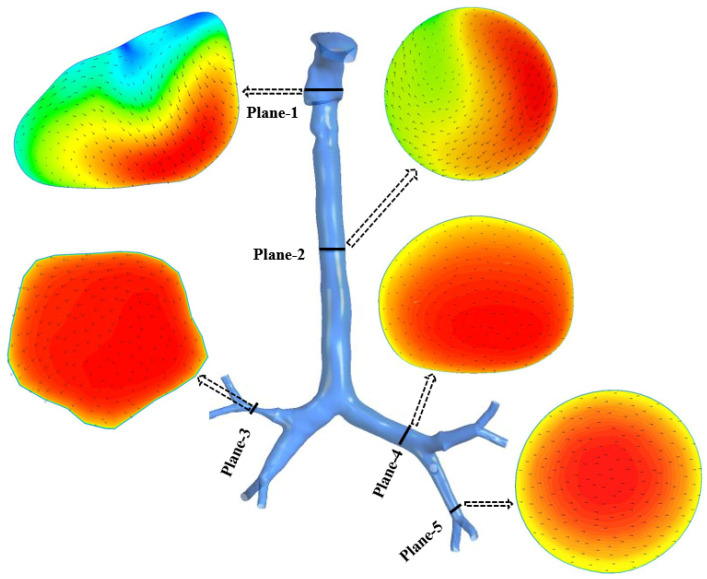
Velocity contours at different positions in the mouth–throat and tracheobronchial airways at a flow rate of 60 L/min.

Figure 14 shows the airflow velocity contours for the stenosis airways at various places, at a flow rate of 60 L/min. Because the anatomical variations and shapes of the stenosis influence the flow patterns, a considerable velocity difference has been detected in the stenosis section of the two models. The airflow velocity contours are affected by pressure-driven force, a significant change in airway curvature, the asymmetric airway shape, and turbulence fluctuation at the stenosis region (Plane-1). It can be observed that 83% of velocity increases at the stenosis section (Plane-3) compared to the healthy lung model. The stenosis lungs showed widely divergent airflow velocity contours at planes 2, 4, and 5, respectively.

**Figure 15:**
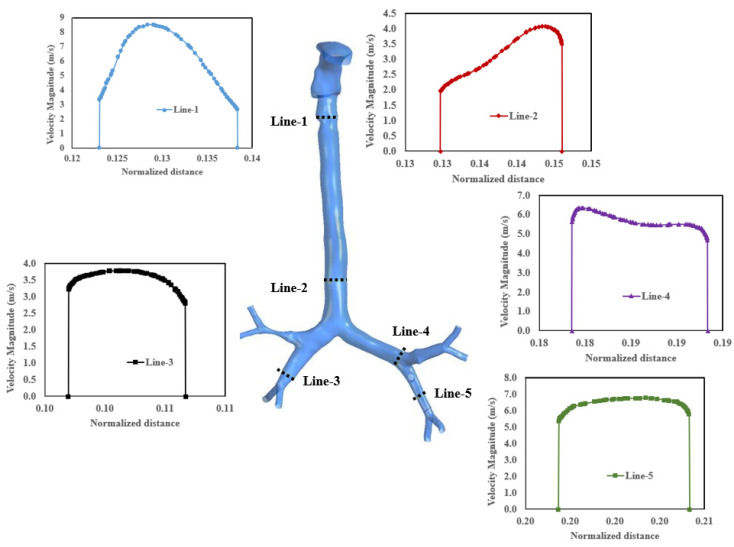
Velocity profiles at different cross-sections in the mouth–throat and tracheobronchial airways at a flow rate of 60 l/min.

Figure 15 shows the velocity profiles on various cross-sections at a flow rate of 60 L/min. Because of the more complex geometry of airways and the separation of flow and secondary vortices created, the velocity profiles are less uniform (line-2 and line-4). However, the velocity is uniformly distributed at the end of the airways (line-3 and line-5).

##### Particle Deposition Efficiency

**Figure 16:**
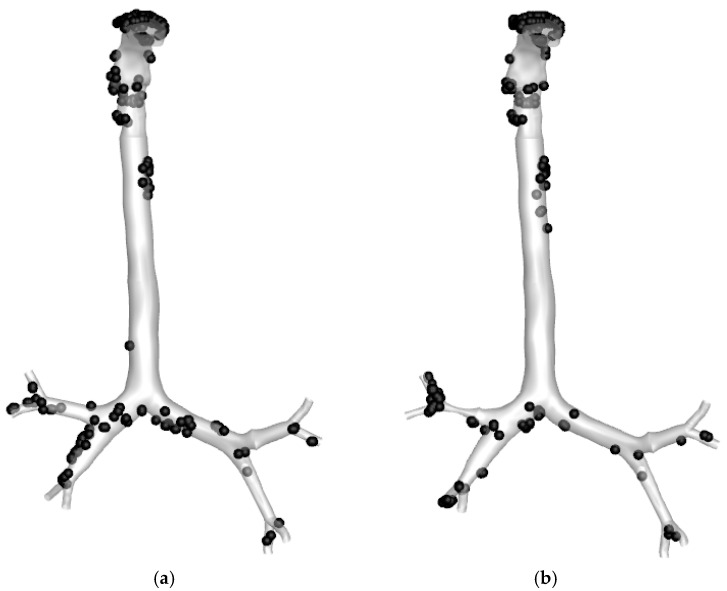
Particle deposition of 2.5 µm particles at a flow rate of 60 L/min; (**a**) Healthy lung model, (**b**) Stenosis lung airways.

Figure 16 shows the overall particle deposition in the stenosis and without stenosis lung mode at a flow rate of 60 L/min. The majority of particles are deposited in the mouth–throat area of the upper airway’s lung. The mouth–throat shape is an irregular and complicated form. The resulting dynamic behaviour impacts when particles cross the stenosis section, their velocity increases, and they collide with the bifurcation wall. Therefore, the higher velocity impacted the particle trajectory, and the dramatic shift in airway curvature increased the deposition at the bifurcation area. As a result, the deposition in the stenosis lung model is higher than in the healthy lung model. More specially, the total deposition of the particle in the stenosis model and healthy lung model are 13.94% and 14.48%, respectively.

## 4. Discussions

The study analysed the average and maximum temperatures for the selected regions. The overall analysis of the maximum and average temperature reports that Sydney Central East and South East are the hottest regions compared to the other selected regions during the first week of November. The overall monthly average temperature data shows that January is the hottest month in 2019–2020, and the Upper Hunter region is the hottest place during the bushfire season. The maximum monthly temperature has an upward trend from October to January, and a considerable decrease occurred from January to June. The Upper Hunter region has the lowest temperature compared with the other regions. The lowest average monthly wind speed is reported in the Upper Hunter region, which may influence the higher temperature in this region. The monthly average NO emission at Upper Hunter consistently maintained the highest value compared to the other selected locations. The monthly average NO emission dramatically rises from March to June in other selected regions. The highest monthly average NO_2_ emission also occurs between May and June among these selected regions during the same periods, except in Sydney South West and Sydney North West. The monthly average PM_10_ emission from December to February is in considerably decline and it turns to become slowly drop from February to June during 2018–2019 and 2019–2020. There is a notable decrease in daily average PM_2.5_ emissions occurs between the end of December and the beginning of January. The highest average PM_2.5_ emissions of 45 µg/m^3^ were found in Sydney South West in December 2019–2020. This month’s average PM_2.5_ emissions from other selected locations were around 23–26 µg/m^3^. The crude rate of Respiratory Conditions in 2019–2020 became higher than the same data in the previous 5 years, from the middle of November to the beginning of January. During these two periods: the beginning of September to the beginning of November and the end of January to the end of February, crude rate of Respiratory Conditions in 2019–2020 is lower than same data in the previous 5 years. For Asthma, the crude rate before the bushfire period (2019–2020) is lower than the crude rate of the previous 5 years period. However, the health data reports a higher crude rate for the asthma patient during the bushfire period (2019–2020) than in the previous 5 years. The increase of asthma patients during the bushfire period indicates the impacts of the bushfire smoke and exhaust particles on respiratory health [1]. In terms of COPD (acute exacerbation), the crude rate in 2019–2020 becomes 1.1–1.2 times higher than the same date in previous 5 years, from the end of October to the end of February. The hospitalisations were more severe in 2019–2020, especially with the peak increase in respiratory diseases and Asthma concentrated from the end of November to the end of January. According to the previous figures and tables discussion, the information can be confirmed that pollutants produced by bushfires and changes in pollutants driven by heat waves greatly impact human respiratory health, directly leading to a significant gain in human respiratory diseases [48,49,50,51,52,53,54,55,56]. The crude rate of Chest Pain, and Burns and Dehydration in 2019–2020 is respectively 1.1–1.2 times and 1.0–1.3 times less than the same data in previous 5 years, which shows the crude rate follows the decreasing trend. To understand the trend of selected heart conditions, cerebrovascular conditions, and mental health issues in 2019–2020 and 5 years ago, it is important to consider all the variables, such as the increased CO and ozone emission due to bushfire, the decreased amount of PM_2.5_ and PM_10_ particulate matter due to heavy rains and floods, and the fluctuations in air quality due to epidemics. Air quality is inextricably linked to human health; therefore, the study of air quality research should be widely paid attention to. Some limitations of the study are listed as follows:

- The study analysed the air-quality data. However, a comprehensive statistical analysis is not considered for the present study. The period of several previous years will be considered in the future study for selected substances.
- The study did not analyse the hourly data during the peak bushfire periods;
- The study did not consider the bushfire data for other regions of Australia, limiting only to NSW;
- No prediction model is proposed for the air quality, which will be developed in the future study;
- The relationship between humidity and temperature will be considered in future study.

## 5. Conclusions

The present study critically analysed New South Wales’s air quality and corresponding health impacts based on Heat Wave and Bushfire Meteorology. The study also analysed the health data in 2019–2020 and the previous 5 years. The key findings from this study are as follows:

- The analysis reports that the Upper Hunter region is the hottest place compared to the other selected regions during the bushfire period.
- The monthly average Ozone in Sydney South West is higher than in other regions.
- The monthly average NO emission in the Upper Hunter region in 2018–2019 and 2019–2020 is higher than in the regions.
- The monthly average PM_10_ emission during the bushfire period (2019–2020) in the Upper Hunter region is higher than in other areas, and the opposite scenario is observed for the previous year (2018–2019).
- The CO, and PM_2.5_ emission during the four-month period of bushfire in 2019–2020 is much higher in all regions than in 2018–2019.
- The number of respiratory diseases in 2019–2020 from October to February is higher than the same data in the previous 5 years.
- PM_2.5_ particles have the ability to penetrate deep into the lungs. After generation G3, it is expected that 85.52% of the particle will reach the deep lung.

The findings of this study and along with more analysis would improve the knowledge of the heat wave and bush fire meteorological variable’s impacts on air quality. The future study would employ an innovative machine learning approach to analyse and predict heat wave and bush fire meteorology accurately.

## Figures and Tables

**Table 1:** Data collection points at various locations of Sydney Central East, North West, South West, and Upper Hunter.

Sydney Central East	Sydney North West	Sydney South West	Upper Hunter
1. Cook and Phillip	1. Paramatta North	1. Bargo	1. Singleton
2. Randwick	2. Richmond	2. Bringelly	2. Camberwell
3. Rozelle	3. St Marys	3. Cambden	3. MuswellBrook
4. Chullora	4. Vineyard	4. Campbelltown	4. Maison Dieu
5. Earlwood	5. Prospect	5. Liverpool	5. Mount Thorley
6. Macquarie Park	6. Rouse Hill	6. Macarthur	6. Wybong
7. Lindfield	-	7. Oakdale	7. Bulga
-	-	-	8. Aberdeen
-	-	-	9. Merriwa
-	-	-	10. Warkworth

**Table 2:** Maximum CO [ppm] emission for a wide range of periods in different areas of Sydney.

Month	Sydney Central East 2018–2019	Sydney Central East 2019–2020	Sydney North West 18–19	Sydney North West 2019–2020	Sydney South West 2018–2019	Sydney South West 2019–2020
November	0.199	0.201	0.092	0.227	0.189	0.245
December	0.144	0.260	0.084	0.276	0.206	0.416
January	0.186	0.324	0.100	0.299	0.237	0.431
February	0.144	0.149	0.066	0.133	0.184	0.201
March	0.165	0.138	0.101	0.118	0.165	0.154
April	0.190	0.146	0.147	0.145	0.194	0.188
May	0.220	0.167	0.190	0.197	0.258	0.190
June	0.254	0.200	0.224	0.253	0.237	0.209

**Table 3:** Number and crude rate of admitted patient hospitalisations during the 2019–2020 bushfire season and previous 5-years average.

	Respiratory Conditions				Asthma				COPD (Acute Exacerbation)				Breathing Abnormalities			
	2019–20		5-Years (Avg)		2019–20		5-Years (Avg)		2019–20		5-Years (Avg)		2019–20	5-Years (Avg)		
Week	*n*	Crude Rate	*n*	Crude Rate	*n*	Crude Rate	*n*	Crude Rate	*n*	Crude Rate	*n*	Crude Rate	*n*	Crude Rate	*n*	Crude Rate
1–7 September	3559	44.0	3676.6	47.5	224	2.8	235.8	3.1	124	1.5	140.2	1.8	265	3.3	194.2	2.5
8–14 September	3323	41.1	3608.0	46.6	178	2.2	229.2	3.0	151	1.9	141.6	1.8	282	3.5	191.8	2.5
15–21 September	3320	41.1	3410.0	44.0	199	2.5	219.8	2.8	154	1.9	143.0	1.8	247	3.1	190.2	2.5
22–28 September	3261	40.3	3276.6	42.3	200	2.5	220.8	2.9	132	1.6	128.2	1.7	258	3.2	198.6	2.6
29 September–5 October	3198	39.5	2935.4	37.9	184	2.3	189.4	2.4	122	1.5	134.4	1.7	267	3.3	187.2	2.4
6–12 October	2909	36.0	2963.6	38.3	175	2.2	182.4	2.4	127	1.6	146.8	1.9	207	2.6	197.0	2.5
13–19 October	2887	35.7	2814.4	36.4	166	2.1	200.2	2.6	137	1.7	131.6	1.7	271	3.4	200.0	2.6
20–26 October	2956	36.6	2778.8	35.9	193	2.4	239.0	3.1	154	1.9	127.8	1.6	255	3.2	203.8	2.6
27 October–2 November	2863	35.4	2813.0	36.3	215	2.7	250.4	3.2	164	2.0	126.2	1.6	244	3.0	218.4	2.8
3–9 November	2856	35.3	2817.4	36.4	217	2.7	236.2	3.1	163	2.0	129.0	1.7	206	2.5	199.8	2.6
10–16 November	2829	35.0	2734.8	35.3	233	2.9	209.0	2.7	173	2.1	127.0	1.6	236	2.9	191.0	2.5
17–23 November	2942	36.4	2708.4	35.0	263	3.3	208.8	2.7	183	2.3	139.4	1.8	251	3.1	201.0	2.6
24–30 November	2871	35.5	2633.0	34.0	208	2.6	203.6	2.6	167	2.1	130.4	1.7	247	3.1	191.6	2.5
1–7 December	2943	36.4	2692.4	34.8	240	3.0	196.6	2.5	196	2.4	125.6	1.6	269	3.3	216.2	2.8
8–14 December	2867	35.5	2607.2	33.7	253	3.1	193.4	2.5	179	2.2	116.0	1.5	263	3.3	204.2	2.6
15–21 December	2630	32.5	2378.0	30.7	229	2.8	198.2	2.6	177	2.2	116.8	1.5	214	2.6	201.6	2.6
22–28 December	2021	25.0	1835.4	23.7	168	2.1	176.4	2.3	143	1.8	113.4	1.5	146	1.8	135.4	1.7
29 December–4 January	2127	26.3	1858.8	24.0	166	2.1	133.4	1.7	182	2.3	117.8	1.5	135	1.7	113.2	1.5
5–11 January	2333	28.8	2066.6	26.7	176	2.2	134.6	1.7	183	2.3	111.2	1.4	164	2.0	146.4	1.9
12–18 January	2306	28.5	2239.8	28.9	158	2.0	136.2	1.8	151	1.9	119.6	1.5	188	2.3	171.6	2.2
19–25 January	2479	30.7	2276.2	29.4	146	1.8	148.6	1.9	159	2.0	121.8	1.6	252	3.1	192.0	2.5
26 January–1 February	2312	28.6	2151.4	27.8	140	1.7	171.6	2.2	125	1.5	107.8	1.4	254	3.1	203.2	2.6
2–8 February	2409	29.8	2412.2	31.2	168	2.1	247.2	3.2	147	1.8	109.0	1.4	259	3.2	244.4	3.1
9–15 February	2580	31.9	2593.6	33.5	220	2.7	351.0	4.5	149	1.8	115.2	1.5	323	4.0	276.4	3.6
16–22 February	2582	31.9	2599.4	33.6	253	3.1	313.6	4.1	120	1.5	117.4	1.5	317	3.9	269.6	3.5
23 February–1 March	2701	33.4	2603.4	33.6	239	3.0	272.0	3.5	132	1.6	107.0	1.4	306	3.8	272.8	3.5

**Table 4:** Number and crude rate of admitted patient hospitalisations during the 2019–2020 bushfire season and previous 5-years average.

	Selected Heart Conditions				Cerebrovascular Conditions				Chest Pain				Mental Health				Burns				Dehydration			
	2019–20		5-Years (Avg)		2019–20		5-Years (Avg)		2019–20		5-Years (Avg)		2019–20		5-Years (Avg)		2019–20		5-Years (Avg)		2019–20		5-Years (Avg)	
Week	*n*	Crude Rate	*n*	Crude Rate	*n*	Crude Rate	*n*	Crude Rate	*n*	Crude Rate	*n*	Crude Rate	*n*	Crude Rate	*n*	Crude Rate	*n*	Crude Rate	*n*	Crude Rate	*n*	Crude Rate	*n*	Crude Rate
1–7 September	2610	32.3	2523.8	32.6	513	6.3	495.6	6.4	675	8.3	711.4	9.2	3124	38.6	2988.2	38.6	49	0.6	52.0	0.7	47	0.6	50.8	0.7
8–14 September	2645	32.7	2578.6	33.3	500	6.2	494.8	6.4	703	8.7	742.6	9.6	3185	39.4	3058.2	39.5	32	0.4	51.8	0.7	46	0.6	47.8	0.6
15–21 September	2755	34.1	2510.8	32.4	510	6.3	495.2	6.4	747	9.2	727.8	9.4	3122	38.6	3016.6	38.9	36	0.4	51.0	0.7	46	0.6	48.4	0.6
22–28 September	2733	33.8	2388.6	30.8	494	6.1	489.8	6.3	745	9.2	701.0	9.1	3222	39.8	2962.8	38.2	44	0.5	41.4	0.5	40	0.5	47.4	0.6
29 September–5 October	2652	32.8	2195.8	28.4	490	6.1	431.2	5.6	683	8.4	692.6	9.0	3122	38.6	2612.0	33.8	39	0.5	52.8	0.7	37	0.5	47.4	0.6
6–12 October	2347	29.0	2441.2	31.5	440	5.4	495.4	6.4	711	8.8	736.2	9.5	2810	34.7	3003.8	38.8	35	0.4	44.8	0.6	42	0.5	45.6	0.6
13–19 October	2755	34.1	2454.0	31.7	581	7.2	493.2	6.4	734	9.1	739.2	9.6	3412	42.2	3113.4	40.2	33	0.4	44.0	0.6	39	0.5	46.4	0.6
20–26 October	2618	32.4	2495.6	32.2	508	6.3	511.0	6.6	692	8.6	732.8	9.5	3410	42.2	3121.6	40.3	38	0.5	45.6	0.6	53	0.7	49.4	0.6
27 October–2 November	2694	33.3	2476.2	32.0	552	6.8	483.4	6.2	714	8.8	747.6	9.7	3552	43.9	3114.4	40.2	29	0.4	48.6	0.6	42	0.5	56.2	0.7
3–9 November	2522	31.2	2452.4	31.7	557	6.9	481.2	6.2	650	8.0	795.8	10.3	3511	43.4	3130.0	40.4	42	0.5	47.2	0.6	51	0.6	47.6	0.6
10–16 November	2539	31.4	2486.8	32.1	552	6.8	482.8	6.2	636	7.9	778.8	10.1	3356	41.5	3127.2	40.4	40	0.5	43.2	0.6	49	0.6	51.4	0.7
17–23 November	2576	31.9	2450.0	31.6	558	6.9	477.2	6.1	679	8.4	778.0	10.1	3433	42.4	3106.0	40.1	37	0.5	48.4	0.6	56	0.7	49.8	0.6
24–30 November	2667	33.0	2516.2	32.5	508	6.3	476.0	6.1	672	8.3	784.8	10.2	3454	42.7	3064.0	39.6	27	0.3	46.8	0.6	49	0.6	55.8	0.7
1–7 December	2577	31.9	2508.4	32.4	532	6.6	472.6	6.1	721	8.9	761.6	9.8	3419	42.3	3103.4	40.1	30	0.4	42.6	0.6	47	0.6	62.4	0.8
8–14 December	2655	32.8	2543.6	32.8	506	6.3	493.4	6.4	728	9.0	728.2	9.4	3311	40.9	3057.8	39.5	35	0.4	46.6	0.6	43	0.5	52.6	0.7
15–21 December	2574	31.8	2454.0	31.7	500	6.2	459.2	5.9	677	8.4	763.4	9.9	3020	37.3	2657.4	34.3	49	0.6	48.0	0.6	68	0.8	58.2	0.8
22–28 December	1353	16.7	1468.8	19.0	326	4.0	308.6	4.0	558	6.9	624.6	8.1	1302	16.1	1356.8	17.5	36	0.4	40.6	0.5	47	0.6	45.4	0.6
29 December–4 January	1524	18.8	1536.0	19.8	337	4.2	321.4	4.1	600	7.4	702.2	9.1	1390	17.2	1508.4	19.5	65	0.8	48.0	0.6	62	0.8	50.0	0.6
5–11 January	1928	23.8	1861.4	24.0	515	6.4	436.6	5.6	700	8.7	725.0	9.4	2772	34.3	2368.6	30.6	42	0.5	41.6	0.5	64	0.8	64.6	0.8
12–18 January	2254	27.9	2057.8	26.6	500	6.2	460.0	5.9	687	8.5	738.0	9.5	3009	37.2	2711.4	35.0	37	0.5	40.2	0.5	41	0.5	73.6	0.9
19–25 January	2425	30.0	2157.8	27.9	539	6.7	478.6	6.2	705	8.7	751.8	9.7	3112	38.5	2770.8	35.8	30	0.4	41.2	0.5	48	0.6	65.2	0.8
26 January–1 February	2175	26.9	2070.2	26.7	486	6.0	436.0	5.6	633	7.8	740.4	9.6	2938	36.3	2585.8	33.4	30	0.4	45.0	0.6	64	0.8	58.4	0.8
2–8 February	2362	29.2	2369.6	30.6	568	7.0	490.4	6.3	645	8.0	764.0	9.9	3290	40.7	3080.6	39.8	49	0.6	46.0	0.6	56	0.7	53.4	0.7
9–15 February	2591	32.0	2378.6	30.7	583	7.2	491.8	6.3	666	8.2	766.8	9.9	3385	41.9	3113.0	40.2	36	0.4	49.4	0.6	32	0.4	57.8	0.7
16–22 February	2585	32.0	2431.6	31.4	582	7.2	497.6	6.4	688	8.5	768.8	9.9	3385	41.9	3113.8	40.2	29	0.4	48.8	0.6	43	0.5	47.2	0.6
23 February–1 March	2531	31.3	2412.4	31.2	575	7.1	511.8	6.6	670	8.3	779.4	10.1	3382	41.8	3274.0	42.3	34	0.4	46.2	0.6	39	0.5	50.2	0.6

## Data Availability

Data will be available upon reasonable request.

## Supplementary Materials

The following supporting information can be downloaded at: https://www.mdpi.com/article/10.3390/ijerph191610388/s1, Table S1: Maximum NO [pphm] emission for a wide range of periods in different areas of Sydney; Table S2: Maximum ozone [pphm] emission for a wide range of periods in different areas of Sydney; Table S3: Monthly maximum PM_10_ emission [µm/m^3^] on different parts of NSW over a wide range of periods; Table S4: Monthly maximum PM_2.5_ emission [µm/m^3^] on different parts of NSW over a wide range of periods; Figure S1: (a) Realistic lung model from mouth to the 3rd generation (b) Ten-layer inflection in the mouth inlet (c) Zoomed-in view on the mouth part (d) Zoomed-in view on the left side of the lung; Figure S2: The average solar at Sydney Central East, North West, South West and Upper Hunter region for a period of eight months during 2018–2019 and 2019–2020, (a) daily average solar, and (b) monthly average solar; Figure S3: The monthly average rain at Sydney Central East, North West, South West and Upper Hunter region for a period of eight months during 2018–2019 and 2019–2020.

## References

[1] Graham A.M., Pringle K.J., Pope R.J., Arnold S.R., Conibear L.A., Burns H., Rigby R., Borchers-Arriagada N., Butt E.W., Kiely L. (2021). Impact of the 2019/2020 Australian megafires on air quality and health. GeoHealth.

[2] Phillips S., Wallis K., Lane A. (2021). Quantifying the impacts of bushfire on populations of wild koalas (*Phascolarctos cinereus*): Insights from the 2019/2020 fire season. Ecol. Manag. Restor..

[3] Law B.S., Gonsalves L., Burgar J., Brassil T., Kerr I., O’Loughlin C. (2022). Fire severity and its local extent are key to assessing impacts of Australian mega-fires on koala (*Phascolarctos cinereus*) density. Glob. Ecol. Biogeogr..

[4] Khan S.J. (2021). Ecological consequences of Australian “Black Summer” (2019–2020) fires: A synthesis of Australian Commonwealth Government report findings. Integr. Environ. Assess. Manag..

[5] Dowdy Andrew J. (2020). Seamless climate change projections and seasonal predictions for bushfires in Australia. J. South. Hemisph. Earth Syst. Sci..

[6] Australian Bureau of Meteorology (2019). Tracking Australia’s Climate Through.

[7] NSW Health. https://www.health.nsw.gov.au/environment/air/Pages/faqs.aspx.

[8] Lucas C., Hennessy K., Mills G., Bathols J. (2007). Bushfire weather in southeast Australia: Recent trends and projected climate change impacts. Consultancy Report Prepared for the Climate Institute of Australia.

[9] Delfino R.J., Brummel S., Wu J., Stern H., Ostro B., Lipsett M., Winer A., Street D.H., Zhang L., Tjoa T. (2009). The relationship of respiratory and cardiovascular hospital admissions to the southern California wildfires of 2003. Occup. Environ. Med..

[10] Johnston F., Hanigan I., Henderson S., Morgan G., Bowman D. (2010). Extreme air pollution events from bushfires and dust storms and their association with mortality in Sydney, Australia 1994–2007. Environ. Res..

[11] Naeher L.P., Brauer M., Lipsett M., Zelikoff J.T., Simpson C.D., Koenig J.Q., Smith K.R. (2007). Woodsmoke health effects: A review. Inhal. Toxicol..

[12] Black C., Tesfaigzi Y., Bassein J.A., Miller L.A. (2017). Wildfire smoke exposure and human health: Significant gaps in research for a growing public health issue. Environ. Toxicol. Pharmacol..

[13] Liu J.C., Pereira G., Uhl S.A., Bravo M.A., Bell M.L. (2015). A systematic review of the physical health impacts from non-occupational exposure to wildfire smoke. Environ. Res..

[14] Borchers Arriagada N., Palmer A.J., Bowman D.M.J.S., Morgan G.G., Jalaludin B.B., Johnston F.H. (2020). Unprecedented smoke-related health burden associated with the 2019–2020 bushfires in eastern Australia. Med. J. Aust..

[15] Larpruenrudee P., Surawski N.C., Islam M.S. (2022). The Effect of Metro Construction on the Air Quality in the Railway Transport System of Sydney, Australia. Atmosphere.

[16] Dennekamp M., Abramson M.J. (2011). The effects of bushfire smoke on respiratory health. Respirology.

[17] Islam M.S., Paul G., Ong H.X., Young P.M., Gu Y.T., Saha S.C. (2020). A Review of Respiratory Anatomical Development, Air Flow Characterization and Particle Deposition. Int. J. Environ. Res. Public Health.

[18] Russell A.G., Brunekreef B. (2009). A Focus on Particulate Matter and Health. Environ. Sci. Technol..

[19] Singh P., Raghav V., Padhmashali V., Paul G., Islam M.S., Saha S.C. (2020). Airflow and Particle Transport Prediction through Stenosis Airways. Int. J. Environ. Res. Public Health.

[20] Berg E.J., Robinson R.J. (2011). Stereoscopic particle image velocimetry analysis of healthy and emphysemic alveolar sac models. J. Biomech. Eng..

[21] Larpruenrudee P., Islam M.S., Paul G., Paul A.R., Gu Y.T., Saha S.C. (2021). Model for Pharmaceutical aerosol transport through stenosis airway. Handb. Lung Target. Drug Deliv. Syst..

[22] Islam M., Gu Y., Farkas A., Paul G., Saha S. (2020). Helium–Oxygen Mixture Model for Particle Transport in CT-Based Upper Airways. US Natl. Libr. Med. Natl. Inst. Health.

[23] Islam M., Saha S., Sauret E., Ong H., Yong P., Gu Y. (2021). Euler–Lagrange approach to investigate respiratory anatomical shape effects on aerosol particle transport and deposition. Toxicol. Res. Appl..

[24] Ma L., Qi H., Sun Z. (2020). Research progress on aerosol particle size distribution characteristics and respiratory system exposure assessment. Acta Sci. Circumstantiae.

[25] Laumbach R., Kipen H. (2012). Respiratory health effects of air pollution: Update on biomass smoke and traffic pollution. Natl. Cent. Biotechnol. Inf..

[26] Islam M.S., Larpruenrudee P., Paul A.R., Paul G., Gemci T., Gu Y., Saha S.C. (2021). SARS-CoV-2 aerosol: How far it can travel to the lower airways?. J. Phys. Fluids.

[27] Kumar P., Morawska L., Birmili W., Paasonen P., Hu M., Kulmala M., Harrison R.M., Norford L., Britter R. (2014). Ultrafine particles in cities. Environ. Int..

[28] Wang H., Reponen T., Lee S.A., White E., Grinshpun S.A. (2007). Size distribution of airborne mist and endotoxin-containing particles in metalworking fluid environments. J. Occup. Environ. Hyg..

[29] Islam M., Saha S., Sauret E., Gu Y., Ristovski Z. (2015). Numerical Investigation of Aerosol Particle Transport and Deposition in Realistic Lung Airway. Proceedings of the 6th International Conference on Computational Methods.

[30] Hendryx M., Islam M.S., Dong G.-H., Paul G. (2020). Air Pollution Emissions 2008–2018 from Australian Coal Mining: Implications for Public and Occupational Health. Int. J. Environ. Res. Public Health.

[31] Kurt O.K., Zhang J., Pinkerton K.E. (2016). Pulmonary health effects of air pollution. Curr. Opin. Pulm. Med..

[32] O’Connor G.T., Neas L., Vaughn B., Kattan M., Mitchell H., Crain E.F., Evans R., Gruchalla R., Morgan W., Stout J. (2008). Acute respiratory health effects of air pollution on children with asthma in US inner cities. J. Allergy Clin. Immunol..

[33] Areal A.T., Zhao Q., Wigmann C., Schneider A., Schikowski T. (2021). The effect of air pollution when modified by temperature on respiratory health outcomes: A systematic review and meta-analysis. Sci. Total Environ..

[34] Watson B.K., Sheppeard V. (2005). Managing respiratory effects of air pollution. Aust. J. Gen. Pract..

[35] Office of Environment and Heritage (2021). Hunter Climate Change Shapshot 2021, NSW Department of Planning & Environment, Sydney. https://www.climatechange.environment.nsw.gov.au/sites/default/files/2021-06/Hunter%20climate%20change%20snapshot.pdf?la=en&hash=50E9A71AA1C43339A1B82C34384D36E1A2C693D2.

[36] Hosker R.P. (1985). Flow around isolated structures and building clusters, a review. ASHRAE Trans..

[37] Pesic D., Zigar D., Anghel I., Glisovic S. (2016). Large Eddy Simulation of wind flow impact on fire-induced indoor and outdoor air pollutuin in an idealised street canyon. J. Wind. Eng. Ind. Aerodyn..

[38] Cichowicz R., Dobrzanski M. (2021). 3D Spatial Analysis of Particulate Matter (PM10, PM2.5 and PM1.0) and Gaseous Pollutants (H_2_S, SO_2_ and VOC) in Urban Areas Surrounding a Large Heat and Power Plant. Energies.

[39] Lateb M., Meroney R.N., Yataghene M., Fellouah H., Saleh F., Boufadel M.C. (2016). On the use of numerical modelling for near-field pollutant dispersion in urban environments—A review. Environ. Pollut..

[40] Atamaleki A., Zarandi S.M., Fakhri Y., Mehrizi E.A., Hesam G., Faramarzi M., Darbandi M. (2019). Estimation of air pollutants emission (PM10, CO, SO_2_ and NOx) during development of the industry using AUSTAL 2000 model: A review method for sustainable development. MethodsX.

[41] Cichowicz R., Dobrzanski M. (2021). Modeling Pollutant Emissions: Influence of Two Heat and Power Plants on Urban Air Quality. Energies.

[42] Weibel E.R. (1963). Morphometry of the Human Lung.

[43] Brook R.D., Rajagopalan S. (2009). Particulate matter, air pollution, and blood pressure. J. Am. Soc. Hypertens..

[44] Solomon P.A., Costantini M., Grahame T.J., Gerlofs-Nijland M.E., Cassee F.R., Russell A.G., Brook J.R., Hopke P.K., Hidy G., Phalen R.F. (2012). Air pollution and health: Bridging the gap from sources to health outcomes: Conference summary. Air Qual. Atmos. Health.

[45] Rahman M.M., Zhao M., Islam M.S., Dong K., Saha S.C. (2021). Aerosol Particle Transport and Deposition in Upper and Lower Airways of Infant, Child and Adult Human Lungs. Atmosphere.

[46] Islam M.S., Larpruenrudee P., Hossain S.I., Rahimi-Gorji M., Gu Y., Saha S.C., Paul G. (2021). Polydisperse aerosol transport and deposition in upper airways of age-specific lung. Int. J. Environ. Res. Public Health.

[47] Rahman M.M., Zhao M., Islam M.S., Dong K., Saha S.C. (2021). Aging effects on airflow distribution and micron-particle transport and deposition in a human lung using CFD-DPM approach. Adv. Powder Technol..

[48] Douglass J.A. (2020). How can air quality affect health?. Intern. Med. J..

[49] Ghosh A., Islam M.S., Saha S.C. (2020). Targeted Drug Delivery of Magnetic Nano-Particle in the Specific Lung Region. Computation.

[50] Cheng Y.-S., Smith S.M., Yeh H.-C., Kim D.-B., Cheng K.-H., Swift D.L. (1995). Deposition of ultrafine aerosols and thoron progeny in replicas of nasal airways of young children. Aerosol Sci. Technol..

[51] Hofmann W. (1982). Dose calculations for the respiratory tract from inhaled natural radioactive nuclides as a function of age–II. Basal cell dose distributions and associated lung cancer risk. Health Phys..

[52] Oakes J.M., Roth S.C., Shadden S.C. (2018). Airflow simulations in infant, child, and adult pulmonary conducting airways. Ann. Biomed. Eng..

[53] Katan J.T., Hofemeier P., Sznitman J. (2016). Computational models of inhalation therapy in early childhood: Therapeutic aerosols in the developing acinus. J. Aerosol Med. Pulm. Drug Deliv..

[54] Haddrell A.E., Lewis D., Church T., Vehring R., Murnane D., Reid J.P. (2017). Pulmonary aerosol delivery and the importance of growth dynamics. Ther. Deliv..

[55] Gemci T., Ponyavin V., Chen Y., Chen H., Collins R. (2008). Computational model of airflow in upper 17 generations of human respiratory tract. J. Biomech..

[56] Loring S.H., Topulos G.P., Hubmayr R.D. (2016). Transpulmonary pressure: The importance of precise definitions and limiting assumptions. Am. J. Respir. Crit. Care Med..

