# Supplementary material for: Heat Wave and Bushfire Meteorology in New South Wales, Australia: Air Quality and Health Impacts
Source: Int J Environ Res Public Health. 2022 Aug 20;19(16):10388. doi: 10.3390/ijerph191610388 (PMC9407765; doi:10.3390/ijerph191610388)
Supplement: Supplementary file 1 [file ijerph-19-10388-s001.zip › ijerph-1808842-supplementary.pdf]

Article

Heat Wave and Bushfire Meteorology in New South Wales, Australia: Air Quality and Health Impacts

Mohammad S. Islam [1,*], Tianxin Fang [1], Callum Oldfield [1], Puchanee Larpruenrudee [1], Hamidreza Mortazavy Beni [2], Md. M. Rahman [3], Shahid Husain [4] and Yuantong Gu [5]

1 School of Mechanical and Mechatronic Engineering, University of Technology Sydney (UTS), 15 Broadway, Ultimo, NSW 2007, Australia
2 Department of Biomedical Engineering, Arsanjan Branch, Islamic Azad University, Arsanjan 6134937333, Iran
3 School of Computing, Engineering, and Mathematics, Western Sydney University, Penrith, NSW 2751, Australia
4 Department of Mechanical Engineering, Zakir Husain College of Engineering & Technology, Aligarh Muslim University, Aligarh 202001, India
5 School of Mechanical, Medical and Process Engineering, Faculty of Engineering, Queensland University of Technology, Brisbane, QLD 4000, Australia
* Correspondence: mohammadsaidul.islam@uts.edu.au

2. Methodology

2.3. Computational Model

By using the modelling software SolidWorks 2019 (DASSAULT SYSTEM Solid-Works Corp., Waltham, MA, USA.), the lung model was created based on the lung dimension from Weibel's model []. The mass and momentum equations were used to calculate the flow and particle transport ($PM_{2.5}$) as the following assumptions:

$$\frac{\partial \rho}{\partial t} + \nabla \cdot (\rho \vec{v}) = S_m \tag{1}$$

where S_m is the mas source term, and

$$\frac{\partial}{\partial t}(\rho \vec{v}) + \nabla \cdot (\rho \vec{v}\vec{v}) = -\nabla \rho + \nabla \cdot \left(\mu \left[(\nabla \vec{v} + \nabla \vec{v}^T) - \frac{2}{3}\nabla \cdot \vec{v} I\right]\right) + \rho \vec{g} + \vec{F} \tag{2}$$

where, ρ is the fluid static pressure, $\rho\vec{g}$ is a body force from gravity, and $\vec{F}$ is a body force from external (particle-fluid interaction) forces.

The k–ϵ turbulence model was selected as a viscous model for this study. The turbulent kinetic energy and dissipation rate in an inertial frame are calculated by the following equations.

For turbulent kinetic energy k,

$$\frac{\partial(\rho k)}{\partial t} + \frac{\partial(\rho k u_i)}{\partial x_i} = \frac{\partial}{\partial x_j}\left[\frac{\mu}{\sigma_k}\frac{\partial k}{\partial x_j}\right] + 2\mu_t E_{ij} E_{ij} - \rho \tag{3}$$

and for dissapation,

$$\frac{\partial(\rho \varepsilon)}{\partial t} + \frac{\partial(\rho \varepsilon u_i)}{\partial x_i} = \frac{\partial}{\partial x_j}\left[\frac{\mu_t}{\sigma_\varepsilon}\frac{\partial \varepsilon}{\partial x_j}\right] + \varepsilon_{1\varepsilon}\frac{\varepsilon}{k} 2\mu_t E_{ij} E_{ij} - \varepsilon_{2\varepsilon}\rho\frac{\varepsilon^2}{k} \tag{4}$$

where u_i is the velocity component in the corresponding direction, E_{ij} is the component of the rate of deformation while μ_t is the eddy viscosity.

For the discrete phase model, air is considered as the primary fluid with the density of 1.225 kg/m^3 and viscosity of 1.7893 × 10^{-5} kg/ms. The pollutant ($PM_{2.5}$) is considered as

the secondary phase with the density of 1100 kg/m[3]. The particles are assumed to be injected from the surface of the mouth area as the inhalation. The 82000 spherical particles are released randomly into the mouth inlet at a time.

The particle transport equation is as follows,

$$\vec{F}_{D,i} = \frac{1}{2} C_D \frac{\pi d_{p,i}^2}{4} \rho(\vec{v}_{p,i} - \vec{v})\left|\vec{v}_{p,i} - \vec{v}\right| \tag{5}$$

where C_D and d_p are the drag coefficient and particle diameter, while $\vec{v}_p$ is the particle velocity. The single particle motion i was modelled by using Newton's second law as follows;

$$\mathrm{m}_{p,i} \frac{\partial \vec{v}_{p,i}}{\partial t} = \vec{F}_{D,i} + \mathrm{m}_{p,i}\vec{g} \tag{6}$$

The particles of diameter 2.5 micron were injected at 60 L/min flow rate. The velocity inlet and outlet flow were taken as the boundary condition. The stationary walls and no-slip conditions were applied. Under the discrete phase model (DPM), wall boundary condition was set as a trap. The pressure-velocity coupling scheme, second-order pressure, and second-order momentum were used for the numerical simulation.

Mesh Generation

The realistic lung model extending from the mouth to the third lung generation is shown in Figure 1a. 4 million tetrahedral volume elements with near-wall prism boundary layers were generated for the grid using ANSYS MESHING to ensure correct CFD results. In addition, ten-layer smooth inflation was used near the walls to guarantee that the boundary layer flow was accurately simulated (Figure 1b). Figures 1c and 1d demonstrate the mesh elements in the side view of the mouth region and left side branches of mesh generation, respectively. Finally, the denser mesh is used at carinal angles to resolve complex flows accurately.

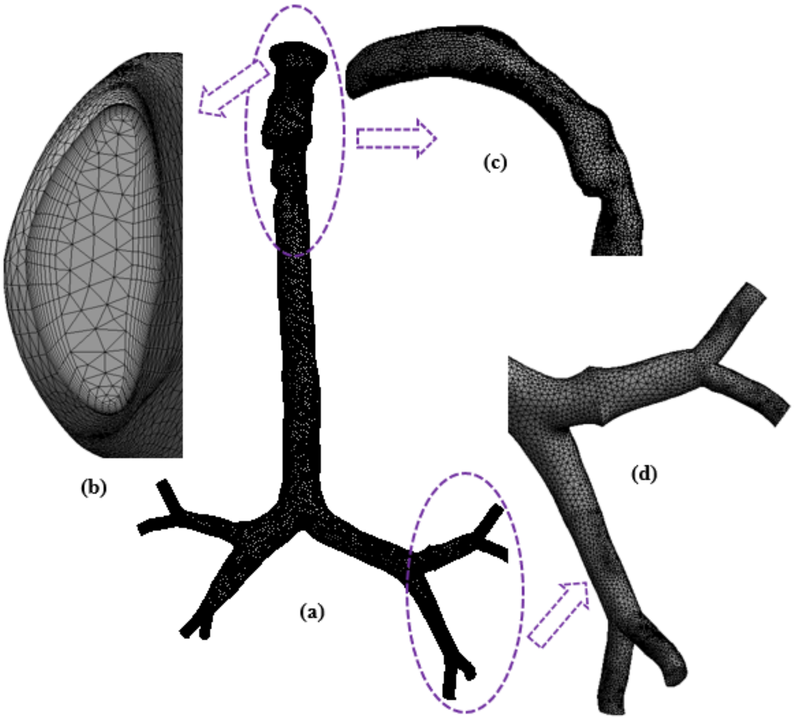

Figure S1. (a) Realistic lung model from mouth to the 3rd generation (b) Ten-layer inflection in the mouth inlet (c) Zoomed-in view on the mouth part (d) Zoomed-in view on the left side of the lung.

3. Results

3.1. Selected Parameters and Pollutants

3.1.1. Average Nitric Oxide (NO) Emission

Table S1. Maximum NO [pphm] emission for a wide range of periods in different areas of Sydney.

Month	Sydney Central East 2018–2019	Sydney Central East 2019–2020	Sydney North West 2018–2019	Sydney North West 2019–2020	Sydney South West 2018–2019	Sydney South West 2019–2020
Nov	4.15	5.23	7.88	4.66	4.72	4.13
Dec	2.57	3.65	5.83	2.78	5.10	5.06
Jan	3.57	4.27	2.78	4.26	2.67	5.00
Feb	6.25	4.37	6.70	5.22	4.25	3.93
Mar	6.20	8.70	6.83	6.70	5.48	6.08
Apr	8.90	5.63	8.68	5.50	5.17	5.48
May	8.05	6.75	10.88	6.02	7.76	6.25
Jun	8.70	11.43	12.20	7.48	7.48	6.63

By contrast, it can be found that the maximum NO emission at Sydney Central East, Sydney North West and Sydney South West in 2019-2020 is respectively 1.2 times, 1.5 times and 1.9 times higher than same data in 2018-2019 during January. By comparison, the maximum NO emission at these three selected regions in 2019-2020 is around 1.1-1.9 times less than the same data in 2018-2019 during February and May. In terms of the maximum NO emission, the data in Sydney North West in 2019-2020 is approximately 1.3-1.8 times less than 2018-2019 expect the data in January. For more analysis, if considering the selected regions in NSW from 2018-2019 to 2019-2020, it can be seen that the maximum NO emission at Sydney North West in 2019-2020 is less than 2018-2019 between November to next year June. However, when it comes to the timeframe, it can be clear seen that the maximum NO emission at Sydney Central East is exponentially affected by the bushfire from November to January, as compared to 2018-2019, the NO emission in 2019-2020 shows an upward trend.

3.1.2. Average Ozone Emission

Table S2. Maximum ozone [pphm] emission for a wide range of periods in different areas of Sydney.

Month	Sydney Central East 2018–2019	Sydney Central East 2019–2020	Sydney North West 2018–2019	Sydney North West 2019–2020	Sydney South West 2018–2019	Sydney South West 2019–2020
**Nov**	4.88	7.50	7.18	9.78	6.23	10.78
**Dec**	6.62	13.13	8.70	12.18	8.72	12.02
**Jan**	7.28	8.36	11.38	**9.64**	9.34	9.80
**Feb**	4.85	7.54	7.13	8.32	8.15	**8.13**
**Mar**	4.85	5.65	5.93	**5.30**	6.80	**5.33**
**Apr**	4.20	4.72	4.65	**4.50**	4.47	5.17
**May**	3.38	**2.80**	4.30	**3.04**	3.80	**3.13**
**Jun**	2.83	2.90	3.08	3.40	3.05	3.17

According to Table 2, it can be seen that the maximum ozone emission in 2019-2020 is always higher than 2018-2019 at November, December and June among these selected regions in NSW. By calculation, the maximum ozone emission at Sydney Central East, Sydney North West and Sydney South West in 2019-2020 is respectively 1.5 times, 1.4 times and 1.7 times higher than same data in 2018-2019 during January. The maximum ozone emission at Sydney Central East, Sydney North West and Sydney South West in

2019-2020 is respectively 1.9 times, 1.4 times and 1.4 times higher than same data in 2018-2019 during December. And for June, which is 1.0 times, 1.1 times and 1.0 times higher than the same data in 2018-2019. However, the maximum ozone emission in 2019-2020 is always less than 2018-2019 during May. From the Table 2, the maximum ozone emission in May in 2019-2020 is respectively 1.2 times, 1.4 times and 1.2 times less than the same data in 2018-2019. In terms of the maximum ozone emission, the data in Sydney Central East in 2019-2020 is always 1.0-1.9 times higher than 2018-2019 expect the data in May.

3.1.3. Average PM_{10} Emission

Table S3. Monthly maximum PM_{10} emission [μm/m^3] on different parts of NSW over a wide range of periods.

Month	Sydney Central East 2018-2019	Sydney Central East 2019-2020	Sydney North West 2018-2019	Sydney North West 2019-2020	Sydney South West 2018-2019	Sydney South West 2019-2020	Upper Hunter 2018-2019	Upper Hunter 2019-2020
Nov	326.07	544.88	417.38	737.18	305.17	586.58	284.9.0	774.39
Dec	112.07	701.28	169.33	819.80	155.58	404.83	59.19	432.37
Jan	99.48	368.72	107.65	867.26	82.78	867.63	61.47	1235.99
Feb	105.86	74.58	100.00	107.82	199.33	89.53	88.55	151.83
Mar	116.15	112.83	120.85	156.44	86.97	97.45	96.62	81.41
Apr	115.88	42.63	60.50	61.44	118.63	53.55	41.27	74.37
May	99.50	34.37	109.70	59.88	86.70	36.72	78.83	37.75
Jun	33.52	44.13	86.22	61.62	43.433	46.23	32.22	55.77

According to Table 3, it can be clearly seen that from November to January, the monthly maximum PM_{10} emission on the various selected regions in NSW during 2018-2019 is always less than the monthly maximum PM_{10} emission during 2018-2019 in previous 3 months. The monthly maximum PM_{10} emission on these four selected regions in November 2019-2020 is approximate 2 times higher than the data in 2018-2019. By contrast, the monthly maximum PM10 emission at Sydney Central East and Upper Hunter in December 2019-2020 is respectively 6.3 times and 7.3 times higher than the same data in 2018-2019. Comparing four different regions in the same month-January, it can be found that the monthly maximum PM_{10} emission at Upper Hunter in 2019-2020 is 20 times higher than the same data in 2018-2019. Compare with the monthly maximum PM_{10} emission in 2018-2019, the fluctuation of monthly maximum PM_{10} emission on these four selected regions in NSW in 2019-2020 is highest during January. In addition, the monthly maximum PM_{10} emission on the four selected regions in May in 2019-2020 is twice less than the same data in 2018-2019. The monthly maximum PM_{10} emission at Sydney North West in 2019-2020 is always higher than the same data in 2018-2019 compared with the other three selected regions expect in May.

3.1.4. Average $PM_{2.5}$ Emission

Table S4. Monthly maximum $PM_{2.5}$ emission [μm/m^3] on different parts of NSW over a wide range of periods.

Month	Sydney Central East 2018-2019	Sydney Central East 2019-2020	Sydney North West 2018-2019	Sydney North West 2019-2020	Sydney South West 2018-2019	Sydney South West 2019-2020	Upper Hunter 2018-2019	Upper Hunter 2019-2020
Nov	43.06	227.47	40.63	404.68	32.97	175.65	53.60	146.97
Dec	25.34	464.26	23.75	530.24	24.54	285.68	19.60	196.10
Jan	25.52	153.08	23.73	236.9	34.55	319.12	26.63	130.93
Feb	20.33	45.50	15.47	66.14	22.87	64.46	25.47	36.90
Mar	33.82	16.55	26.47	18.10	22.02	15.86	20.07	20.23
Apr	91.86	21.22	34.17	28.16	121.86	22.23	24.50	78.40
May	71.32	34.38	66.47	37.74	47.60	25.66	56.53	59.5
Jun	28.30	40.17	37.42	59.12	27.05	26.76	39.70	152.00

By comparison, it can be clearly seen that from November to February, the monthly maximum $PM_{2.5}$ emission on the various selected regions in NSW during 2019-2020 is always higher than the monthly maximum $PM_{2.5}$ emission during 2018-2019. In terms of month, the monthly maximum $PM_{2.5}$ emission at Sydney North West in November 2019-2020 is 9.9 times higher than the same data in 2018-2019 which is the most significant change compared to other three selected regions. According to Table 4, the change trend of $PM_{2.5}$ is most obvious in December compared with the other month, the monthly maximum $PM_{2.5}$ emission at Sydney South West and Upper Hunter in 2019-2020 is 10 times higher than the same data in 2018-2019, and the monthly maximum $PM_{2.5}$ emission at Sydney Central East and Sydney North West in December 2019-2020 is approximately 19 times higher than the same data in 2018-2019. During January, the monthly maximum $PM_{2.5}$ emission at Sydney North West and Sydney South West in 2019-2020 is 10 times higher than 2018-2019.

3.1.5. Average Solar

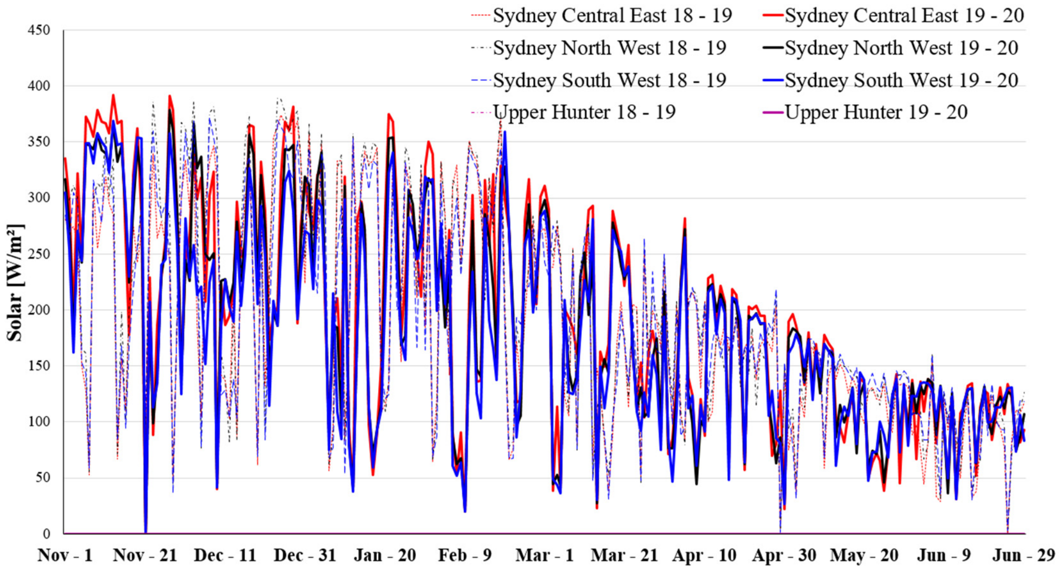

(a)

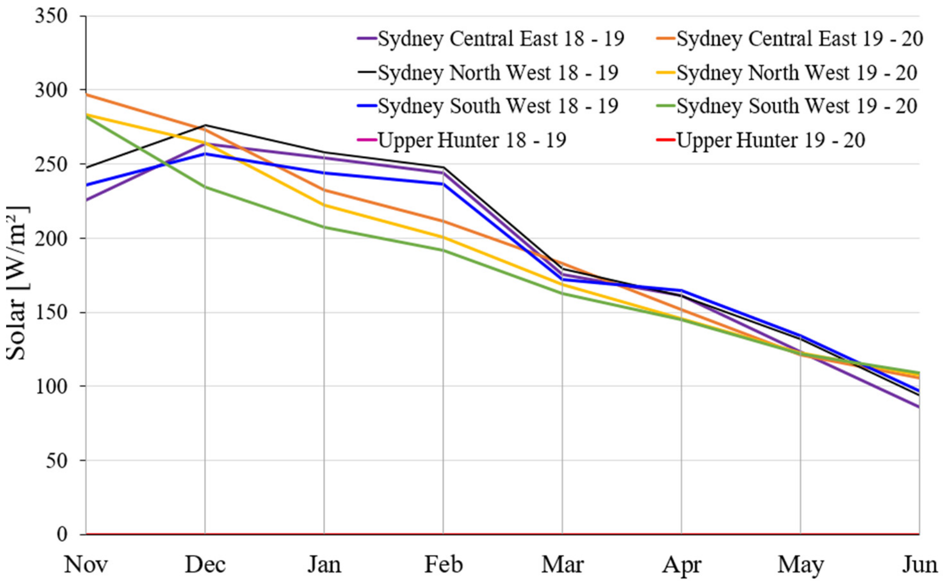

(b)

Figure S2. The average solar at Sydney Central East, North West, South West and Upper Hunter region for a period of eight months during 2018-2019 and 2019-2020, (a) daily average solar, and (b) monthly average solar.

Figure 2 shows the daily average solar and monthly average solar at the various selected locations in NSW for a period of 8 months during 2018-2019 and 2020. Figure 2 (a) shows the overall fluctuation of daily average solar at the four selected locations in NSW within 8 months during 2018-2019 and 2019-2020. The monthly average solar at selected locations from the selected months of these years is presents in Figure 2 (b).

3.1.6. Average Rain

Figure 3 shows monthly average rain at the various selected location in NSW for a period of 8 months during 2018-2019 and 2020. According to this figure, it can be seen that the monthly rain at Sydney Central East is highest in the beginning of February during 2019-2020 compared with the other three locations in NSW, and it also reach the peak in February. Focusing on the same periods for each area, it can be seen that the monthly average rain at Upper Hunter between 2018-2019 and 2019-2020, is usually lower than in other areas in Sydney.

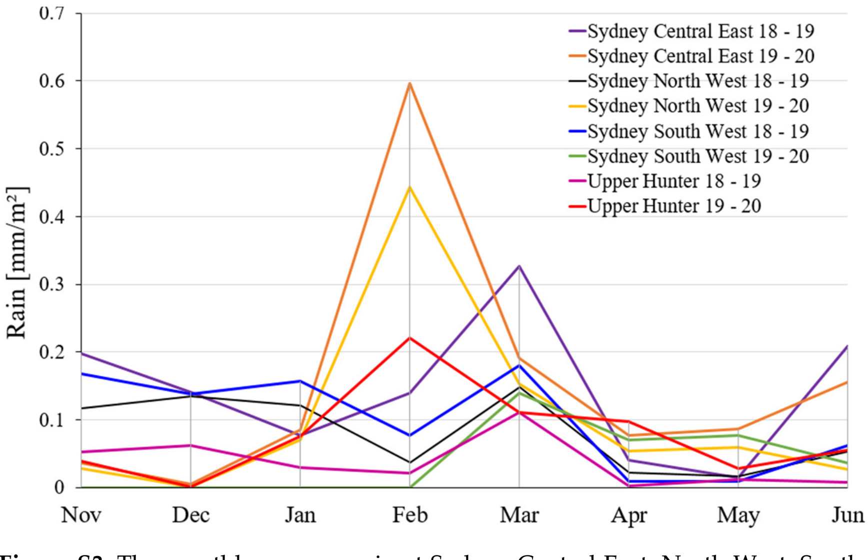

Figure S3. The monthly average rain at Sydney Central East, North West, South West and Upper Hunter region for a period of eight months during 2018-2019 and 2019-2020.

The daily average solar decreased gradually after April and the fluctuation range became smaller from April to June. The average daily solar at Sydney Central East is highest during 2019-2020 among the other three selected locations in NSW. Regarding the comparison in same locations, the monthly average solar during 2019-2020 has a marked downward trend. While, the monthly average solar during 2018-2019 reached the peak in December and went down slowly after December. Compared with the same period during 2018-2019 and 2020, the monthly average solar at Sydney South West is the lowest among the other three selected locations in NSW. In addition, the trend of monthly average rain at Sydney Central East and Sydney North West follows a similar trend, which is the lowest in November and rises significantly from January to February, then experiencing a striking downward trend from February to March. With regards to the comparison in the same selected locations, the monthly average rain during 2018-2019 has a gentle fluctuation and reached its peak in March.
